# Advances in Machine Learning and Deep Learning Algorithm-Assisted Hyperspectral Imaging for Food Quality and Safety Assessment

**DOI:** 10.3390/foods15183287

**Published:** 2026-09-17

**Authors:** Lingwei Hu, Yifan Dong, Chunhong Hu, Mingming Chen, Jitao Li

**Affiliations:** 1 College of Life Science and Agronomy, Zhoukou Normal University, Zhoukou 466001, China; 2 Department of Microelectronics, Jiangsu University, Zhenjiang 212013, China; 3 School of Physics and Telecommunications Engineering, Zhoukou Normal University, Zhoukou 466001, China

**Keywords:** hyperspectral imaging, machine learning, deep learning, food quality, food safety, non-destructive detection

## Abstract

Food quality and safety have become increasingly critical public health concerns, while traditional detection approaches are constrained by laborious sample preparation, lengthy analysis times, and destructive procedures. Hyperspectral imaging (HSI) has emerged as a rapid, non-destructive method that synergistically fuses spatial and spectral information for comprehensive food quality and safety assessment. However, the high dimensionality and nonlinear features of HSI data pose substantial challenges for traditional chemometric models. This review provides a comprehensive overview of recent advances in machine learning (ML) and deep learning (DL) algorithm-assisted HSI food quality and safety detection. We first introduce the fundamentals of HSI technology and its data processing workflow. We then present a comprehensive and non-mathematical introduction to typical ML and DL algorithms, highlighting their respective strengths and trade-offs. Then, we summarize typical applications across various areas, such as fruit and vegetable quality assessment, meat quality evaluation, egg and tea quality detection, moisture content quantification, variety and origin identification, adulteration and additive detection, heavy metal contamination assessment, mold detection, and plant growth monitoring. Finally, we discuss current challenges, such as data bottlenecks, limited model interpretability and generalizability, and barriers to practical applications, and outline future directions toward intelligent, systematic, and practical HSI systems for food quality and safety detection.

## 1. Introduction

**Figure 1:**
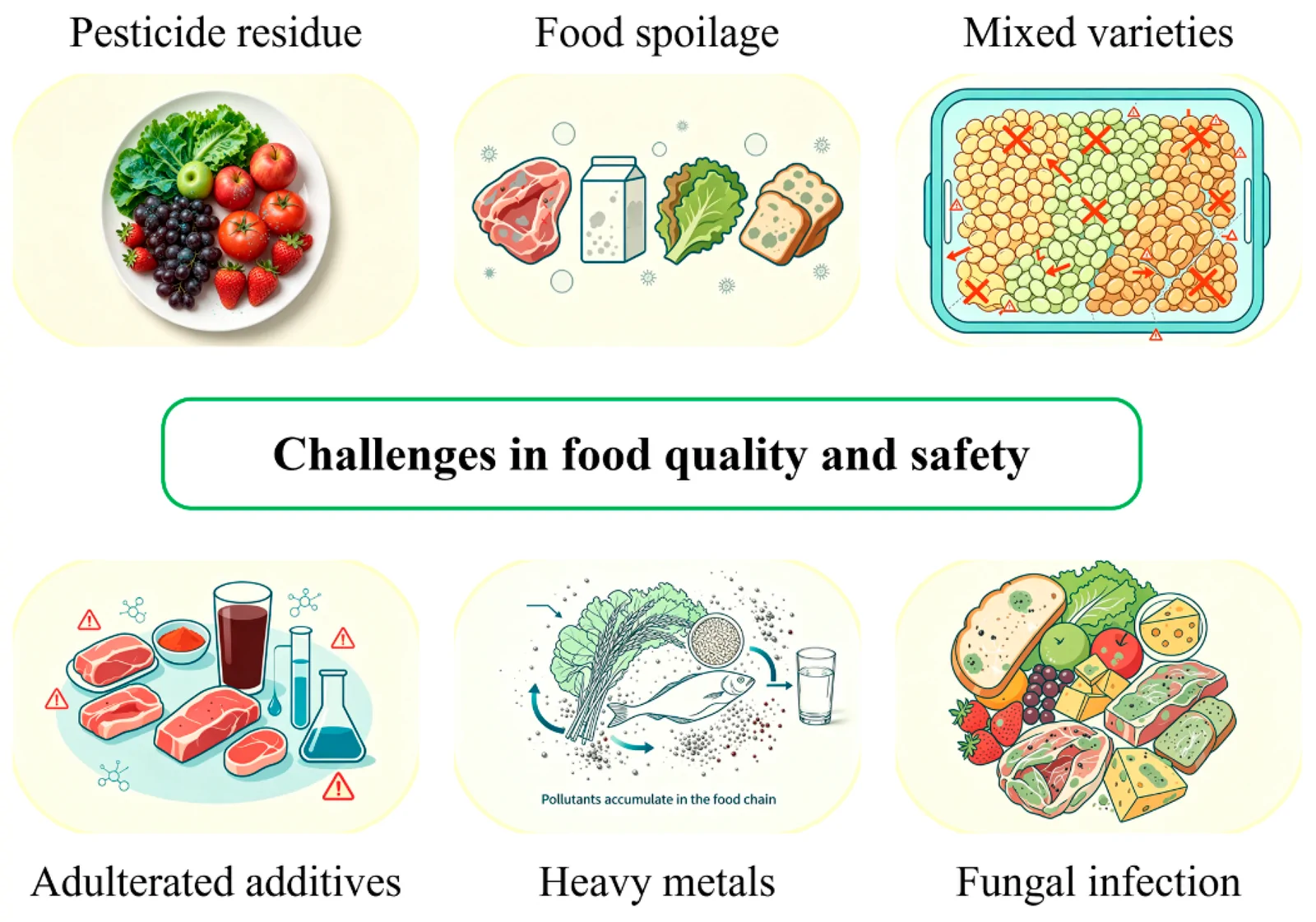
Challenges in food quality and safety.

Food quality and safety have emerged as increasingly essential issues in global public health in recent years. According to the World Health Organization, foodborne illnesses have substantial health and economic impacts [1]. Notably, food products are susceptible to contamination by chemical substances (including pesticide residues, heavy metals, and illegal additives), biological agents (such as parasites and bacteria), and physical hazards at any stage of the supply chain (Figure 1) [2,3,4]. Meanwhile, the progressive globalization of the food supply networks has greatly rendered adulteration schemes as increasingly nuanced and elusive [5]. In general, strict food quality control is critical for protecting human health and promoting economic development. Traditional approaches for assessing food quality and safety, such as high-performance liquid chromatography [6,7,8], gas chromatography [9,10], enzyme-linked immunosorbent assay [11], thin-layer chromatography [12], polymerase chain reaction [13,14], mass spectrometry [9,15,16], and vibrational spectroscopy [17,18], offer high accuracy. However, these methods are hindered by practical shortcomings, including laborious sample preparation, lengthy analysis times, high-skill thresholds, and sample damage. These limitations render them inadequate for the non-destructive and real-time monitoring increasingly demanded by the modern food industry. Consequently, there is an increasing need to explore complementary technologies capable of multi-dimensional information acquisition and rapid response.

Hyperspectral imaging (HSI) technology has recently gained recognition as a rapid and non-destructive technique for food quality detection, with several comprehensive reviews documenting its successful applications in quality grading, moisture analysis, variety and origin authentication, and contaminant detection (additives, heavy metals, and pesticide residues) [19,20,21,22]. The technique uses a focal-plane detector paired with dispersive elements or tunable filters to capture diffuse reflectance [23,24], transmittance [25,26], or fluorescence [27,28] from each sample pixel across the visible-to-infrared spectrum. These measurements are then reconstructed into a three-dimensional data cube that synergistically fuses spatial and spectral information. The spatial dimensions enable the rapid assessment of surface defects and physical damage, whereas the spectral dimension supports chemical analysis based on characteristic wavelengths of organic molecules and functional groups (such as C-N, C=C, and C=O) [29,30]. Despite these significant advantages, substantial challenges remain. A single HSI imaging session generates high-dimensional datasets comprising abundant spectral vectors. Additionally, strong multicollinearity among adjacent spectral bands [31,32,33], along with factors such as surface unevenness and instrument noise, further complicates the HSI data. However, traditional multivariate calibration models, such as principal component regression (PCR) and partial least squares regression (PLSR), often perform poorly at extracting accurate information when analyzing highly nonlinear and mixed HSI data. As a result, it is necessary to explore advanced algorithms to accurately decode HSI data from food samples.

Notably, artificial intelligence, such as machine learning (ML) and deep learning (DL), has proven highly effective for HSI data analysis. In the ML paradigm, researchers often extract physically and chemically meaningful features from HSI data through established feature extraction methods, such as band selection, dimensionality reduction, and wavelet transformation. These features are then fed into shallow models, including support vector machines (SVMs) and random forests (RFs), to build predictive or classification models. This approach is advantageous in scenarios in which spectral physics are well understood, training samples are limited, and high model interpretability is required [34,35]. In contrast, DL methods, particularly convolutional neural networks (CNNs) and their variants, excel at end-to-end translation from the raw spatial–spectral joint matrix to high-level semantic information using multiple layers of nonlinear transformations. This approach effectively avoids the subjectivity and incompleteness inherent in manual feature extraction and has demonstrated superior performance in classification accuracy and feature representation efficiency for estimating soluble solid content (SSC) [36,37] in fruits and detecting illegal adulteration in minced chicken meat [38,39,40]. Notably, these two methodological approaches are not mutually exclusive but rather complementary and synergistic in their evolution. In brief, the former relies primarily on prior domain knowledge to deliver robust baseline solutions and chemical interpretability, while the latter harnesses powerful data-driven fitting capabilities to uncover deep structural patterns embedded within large datasets. It has been reported that the HSI technique combined with DL and ML algorithms facilitates rapid and accurate detection of food quality and safety, as reviewed below.

In fact, ML and DL algorithms have been widely applied across diverse areas, such as computer vision [41], natural language processing [42], intelligent manufacturing [43], smart agriculture [44,45,46,47,48,49,50], and food safety [19,20,21,22]. Fundamentally, ML and DL have demonstrated remarkable versatility in augmenting traditional rule-based methods by learning intrinsic patterns in data, thereby propelling various sectors from digitalization toward intelligent development [51,52]. To accurately extract salient features (i.e., characteristic wavelengths) from HSI data for target detection, various ML and DL algorithms have been extensively explored. Notably, significant advances have been recently made in applying ML and DL algorithms to HSI food quality detection, and the literature on this topic is extensive. However, a comprehensive summary of these developments has yet to emerge. Generally, the existing reviews have broadly surveyed HSI applications in food quality assessment on specific food categories (e.g., grains [21], fruits and vegetables [20]), particular algorithmic paradigms (e.g., DL [20,53,54]), or individual application scenarios (e.g., agriculture [19,55,56], foreign matters detection [57], and foodborne pathogen detection [58]), often without providing a comprehensive and comparative analysis of the fundamental principles, strengths, and trade-offs between traditional ML and DL methodologies for decoding HSI data. To fill this gap, this review presents a detailed overview of the fundamentals of ML and DL algorithms, methodological comparisons, and their typical applications in HSI food quality and safety detection. It is organized as follows: Section 2 shows the fundamentals of HSI technology, such as its basic principles and HSI data processing. Section 3 details the principles of typical ML and DL algorithms. Section 4 presents typical applications of the ML and DL algorithms in HSI food quality and safety assessment, including detecting quality, measuring moisture content, identifying varieties, and detecting alterations, heavy metals, bacterial infections, and harmful substances, across a variety of foods such as fruits, vegetables, meats, grains, eggs, and tea. The data for this review were gathered from the Web of Science database, concentrating on studies published from 2017 to 2026 that involve the topics ‘hyperspectral imaging’ and ‘food quality and safety detection and assessment’. Finally, the review addresses current challenges and prospects for future research and technological progress.

## 2. Fundamentals of Hyperspectral Imaging

**Figure 2:**
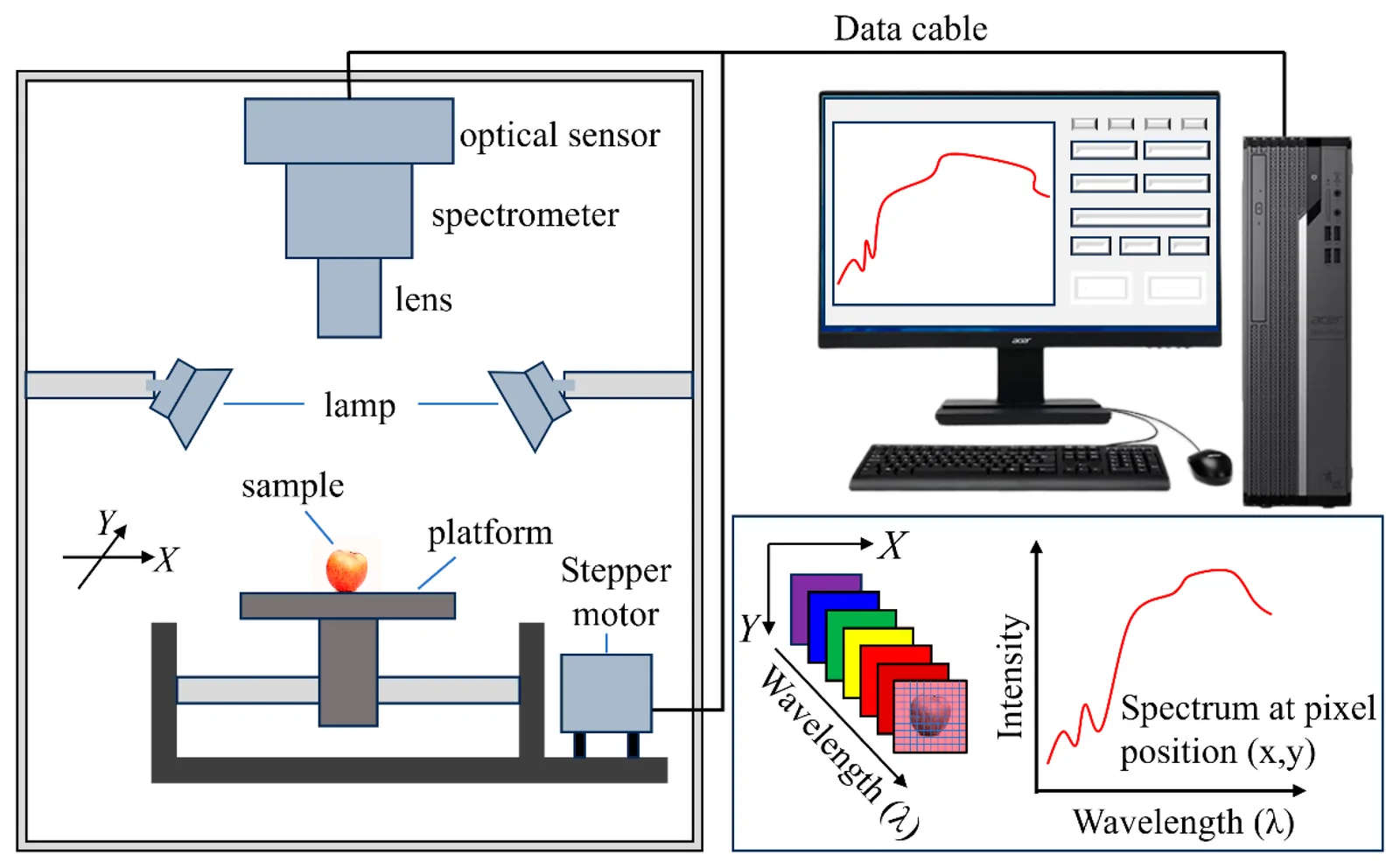
Schematic diagram of the hyperspectral imaging (HSI) system. The inset illustrates the 3D data cube, which comprises 2D spatial information (x, y) and 1D spectral information (λ) [22].

HSI is a detection technology that integrates imaging with spectral analysis. It enables real-time acquisition of detailed spectral and spatial information from food samples and efficiently identifies external and internal features with high sensitivity and accuracy. This capability is rooted in the physical principle that distinct materials exhibit characteristic absorption and reflection signatures at specific wavelengths (i.e., spectral fingerprint) [59]. In practice, HSI captures continuous and narrowband (less than 10 nm) reflectance or fluorescence data at every pixel ranging from the visible-to-infrared spectrum (typically 400–2500 nm), generating a 3D data cube (x, y, λ), where the x and y dimensions encode spatial location and the λ dimension carries detailed spectral information (Figure 2). Owing to high spectral resolution, HSI technology is capable of detect microscopic physical and chemical changes, allowing for precise identification and quantitative analysis of target components.

Figure 2 depicts the typical schematic of an HSI system that integrates optics, mechanics, electronics, and data-processing modules. Briefly, the system comprises optical imaging and spectroscopic units, image sensors, a sample platform, light sources, data acquisition and control systems, and data processing and analysis software [60]. Generally, the optical imaging and spectroscopic units, composed of lenses and dispersive elements including prism gratings or tunable filters, are responsible for spectral decomposition. The image sensors, typically high-sensitivity Si-based CCD/CMOS arrays or InGaAs-based cooled sensors, capture both spatial imagery and spectral data for each pixel across numerous narrow bands. The light source typically utilizes halogen lamps or laser-driven broadband sources to ensure uniform illumination across a wide spectral range. The data acquisition and control systems generally include high-speed interfaces, synchronization controllers, and motion modules that coordinate spectral data collection, spatial scanning, and signal transmission. The software suite offers functions such as radiometric calibration, spectral correction, feature extraction, chemometric modeling, and visualization, and transforms raw data into spectrally resolved images suitable for qualitative and quantitative analysis. Collectively, the entire system integrates the acquisition of raw detector responses with subsequent data processing to generate spectral images [60]. Notably, the optical path can accommodate a microscopy module for high-resolution analysis of small or microscopic samples [61,62,63].

**Figure 3:**
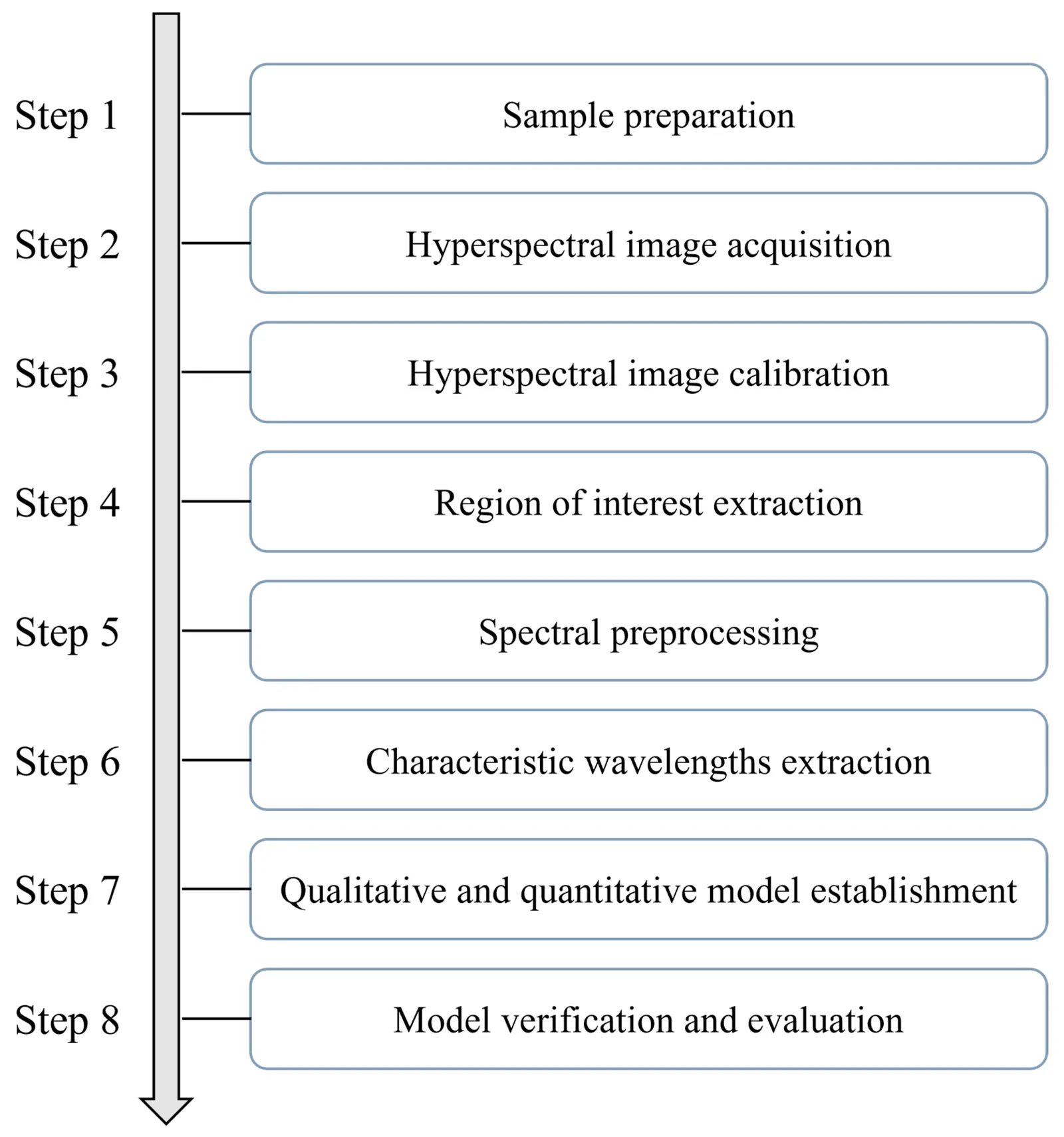
Typical workflow of hyperspectral imaging data processing. DL and ML algorithms are generally applied to Steps 5–7.

In practice, detailed information, such as chemical compositions and contents, is deeply concealed in the HSI images. Figure 3 shows the typical workflow for processing HSI data for food quality evaluation. The quality of HSI images and subsequent data processing is vital for constructing models that accurately reveal the chemical composition and content of food samples [64]. Initially, several parameters, such as spatial and spectral resolution, exposure time, and scan step size, should be optimized during data acquisition to improve the quality of HSI images with a high signal-to-noise ratio. Notably, various types of noise, including electrical noise from the HSI system and environmental noise, can be further suppressed by using methods including Savitzky-Golay (SG) smoothing [65,66], standard normal variate (SNV) [65,67], multiplicative scatter correction (MSC) [62,68], first-derivative (FD) [69,70], and second-derivative (SD) [69,71]. As shown, these techniques are selectively employed to preprocess the HSI data to assess various chemical compositions and their contents in food samples. Additionally, extracting characteristic wavelengths has proven critical for dimensionality reduction and for enhancing the predictive model’s reliability [31,72], which can be achieved by using various approaches such as statistical approaches (e.g., variable importance in projection (VIP) [73,74] and competitive adaptive reweighted sampling (CARS) [75,76]), geometric methods (typically, successive projections algorithm (SPA) [75,77]), and model-stability approaches (e.g., RF [35,78] and SVM [66,79]). Notably, these techniques are often employed in combination rather than independently to achieve high predictive accuracy, as detailed below.

Following characteristic wavelength extraction and dimensionality reduction, a reliable spectral-target mapping is then established, with quantitative regression relying on PLSR [80,81,82], support vector regression (SVR) [80,83], and DL models (such as CNNs [36,84] and LSTM [85,86,87]), and qualitative classification (such as SVM [88,89,90], RF [34,91,92]). To ensure the reliability of the developed models, the dataset is rigorously partitioned into training, validation, and testing sets. Performance evaluation is conducted using several metrics, such as classification accuracy, coefficient of determination (*R*^2^), root mean square error (RMSE), confusion matrices, and residual prediction deviation (RPD).

## 3. Fundamental Concepts of Typical Machine Learning and Deep Learning Algorithms

Recently, HSI technology integrated with AI has demonstrated notable benefits in rapid, non-destructive, and on-site analysis of food quality and safety. Nonetheless, the practical success of this technology hinges on the thoughtful design of preprocessing and feature extraction strategies for each specific application. To inform the methodological choices for HSI food quality and safety detection, this section offers a concise, non-mathematical introduction to typical ML and DL algorithms.

### 3.1. Typical ML Algorithms

**i. Partial Least Squares Regression (PLSR)**. PLSR establishes a linear relationship between a spectral matrix (*X*) and a response matrix (*Y*) by projecting both onto a shared latent subspace, where the extracted components are oriented to maximize the covariance between the *X*-scores and *Y*-scores while preserving variance in the predictor block. Notably, this algorithm addresses the high dimensionality and multicollinearity inherent in HSI data while maintaining robust predictive accuracy [93]. However, its linear approach restricts its capacity to model nonlinear spectral relationships. It is a basic chemometric tool due to its high computational efficiency and interpretability, though it is inherently linear in its standard form. In recent years, the PLSR algorithm has been employed for quantitative prediction of various component contents, such as SSC in fruits [36,94] and moisture content in leaves [95].

**ii. Support Vector Classifier (SVC)**. SVC is a discriminative model that finds the best-separating hyperplane by maximizing the margin between different classes in the feature space. By leveraging kernel methods (e.g., Radial Basis Function (RBF) and polynomial kernels), SVC effectively addresses linear inseparability via implicit nonlinear feature mapping without explicitly computing the transformed coordinates [96]. This makes SVC advantageous for classification problems characterized by limited sample sizes and high-dimensional feature spaces, while also showing moderate resilience to noisy observations when using a soft-margin formulation. In HSI food quality studies, SVC has been widely adopted for qualitative classification, such as food variety and origin identification [27,97], freshness grading [98], and adulteration detection [99,100]. However, its performance is sensitive to kernel selection and hyperparameter tuning, and the training complexity can increase substantially with large-scale datasets.

**iii. Support Vector Regression (SVR)**. SVR operates through developing a regression function that attempts to fit training samples within an *ε*-insensitive tube, while deviations beyond this margin are penalized by introducing slack variables. This algorithm explicitly formulates a trade-off between model flatness and empirical training error, conferring strong generalization ability in small-sample nonlinear problems [101]. This property affords the SVR algorithm a distinct advantage in HSI food quality detection, particularly for the quantification of quality attributes such as SSC in fruits [102,103] and protein content in grains [104]. However, its predictive accuracy depends on the selection of parameters (such as the regularization parameter *C*, tube width *ε*, and kernel parameters), which should be carefully optimized.

**iv. Least Squares Support Vector Machine (LSSVM)**. LSSVM is a variant of the standard support vector machine (SVM). It simplifies the standard SVM formulation by replacing inequality constraints with equality constraints, transforming the quadratic programming problem into a tractable linear system. This modification accelerates model training while maintaining the kernel-based nonlinear mapping capability [105]. Recently, LSSVM has been successfully employed to detect SSC in various fruits achieving R^2^ values higher than 0.90 [89,106]. However, LSSVM loses the sparsity property, as all training samples receive non-zero Lagrange multipliers and consequently serve as support vectors, potentially limiting the model interpretability and scalability in large-sample applications.

**v. Least Squares Support Vector Regression (LSSVR)**. Similar to LSSVM, LSSVR adopts the same formulation by replacing the inequality constraints of standard support vector regression (SVR) with equality constraints, thereby transforming the regression problem into a directly solvable system of linear equations. This reformulation accelerates training while retaining the kernel method’s capacity to model nonlinear relationships [105]. Generally, LSSVR is suited for nonlinear regression tasks involving small- to medium-sized datasets, where its training efficiency can be fully exploited without the scalability constraints becoming prohibitive. Nonetheless, this computational benefit sacrifices the sparse solution property, affecting both model storage and prediction speed.

**vi. K-Nearest Neighbor (KNN)**. The KNN algorithm is a memory-based and instance-driven classification method that does not construct an explicit training model. Instead, it classifies a test sample by identifying the K closest training instances in the feature space and assigning the majority class label among these neighbors. KNN is sensitive to noisy data yet performs robustly when applied in an appropriate feature space [107]. KNN imposes minimal assumptions on data distribution and achieves rapid training with zero optimization cost. However, it is susceptible to the ‘curse of dimensionality’ when applied to high-dimensional data [108]. Consequently, its predictive efficiency and accuracy heavily rely on prior dimensionality reduction.

**vii. Random Forest (RF)**. RF is an ensemble learning algorithm based on decision trees. In brief, it generates multiple training subsets via bootstrap sampling and, at each node split, randomly selects a subset of features for optimal partitioning. The final output is determined by majority voting for classification tasks and by averaging for regression tasks. Notably, this algorithm has shown practical robustness to noisy features and outlier samples that may arise from instrumental noise or sample heterogeneity [109,110]. In addition, RF effectively handles high-dimensional HSI data without severe overfitting and provides intrinsic feature importance rankings, which facilitate the extraction of characteristic wavelengths for model simplification and interpretation [109,110]. However, its performance may degrade when the dataset shows strong linear correlations or if the feature set is too limited.

**viii. Naïve Bayes (NB)**. NB is a probabilistic classifier based on Bayes’ theorem, which assumes conditional independence among features. This allows the joint probability to factorize into a product of per-feature conditional probabilities, thereby enabling efficient posterior probability estimation and class prediction by maximum a posteriori decision. Notably, this algorithm is simple and computationally effective, while the independence assumption is almost invariably violated in HSI data due to strong inter-band correlations [111]. As a result, directly applying NB algorithms to raw HSI data is often discouraged unless effective decorrelation preprocessing (e.g., PCA-based dimensionality reduction) is pre-performed to mitigate the adverse effects of multicollinearity.

**ix. Discriminant Analysis Classifier (DAC)**. DAC is a family of statistical classifiers, with Linear Discriminant Analysis (LDA) and Quadratic Discriminant Analysis (QDA) being the two most common variants for high-dimensional spectral classification tasks. Both methods assume that the feature vectors of each class are drawn from a multivariate normal distribution and perform classification by computing the posterior probability for each class under the Bayesian decision rule. LDA assumes a common covariance matrix for all classes, producing a linear decision boundary, whereas QDA allows each class to have its own covariance matrix, resulting in a quadratic decision boundary. In HSI food safety studies, since HSI data frequently violate the assumption of multivariate normality, the performance of LDA and QDA is limited in classifications involving complex spectral overlap [112]. Furthermore, in typical high-dimensional scenarios where the number of spectral bands exceeds the sample size, the covariance matrices required by both LDA and QDA become singular and cannot be reliably estimated, further compromising their practical utility.

**x. Extreme Gradient Boosting (XGBoost)**. XGBoost is a scalable ensemble method that extends the gradient boosting framework with systematic optimizations. It builds decision trees sequentially, where each new tree is trained to optimize a second-order Taylor expansion of the loss function derived from the previous model’s predictions. By leveraging both first-order gradients and second-order curvature information for tree splitting and leaf weighting, this approach achieves faster convergence and yields higher-quality splits than first-order methods. Notably, XGBoost incorporates L1 and L2 regularization to penalize tree complexity, a strategy particularly valuable in high-dimensional HSI data [113]. These advantages make XGBoost a good choice for HSI food quality assessment, such as fruit quality grading [114] and mycotoxin prediction in grains [115]. XGBoost is generally effective for complex nonlinear regression tasks due to its strong noise resistance. However, it often favors high-frequency features over low-frequency but valuable ones and shows a tendency to overfit training data.

**xi. Extreme Learning Machine (ELM)**. ELM is a single-hidden-layer feedforward neural network. It randomly initializes the input weights and hidden biases and keeps them fixed throughout training, while the output weights are analytically determined via the Moore–Penrose generalized inverse. This non-iterative learning scheme eliminates the need for backpropagation-based parameter tuning and yields a global optimum, making ELM algorithm attractive for rapid model development and prediction of food quality indicators (such as lycopene and SSC) [116,117]. However, the random initialization may lead to performance variability across independent runs owing to the stochastic nature of the feature mapping, and the number of trainable parameters, which scales with the number of hidden neurons, and can become substantial when large networks are employed.

**xii. Principal Component Analysis (PCA)**. PCA algorithm is an unsupervised linear dimensionality reduction technique that projects high-dimensional data onto a lower-dimensional subspace via orthogonal transformation. The resulting principal components are linearly uncorrelated and retain most of the total variance of the original data with a reduced number of variables. In HSI food quality analysis, PCA has been widely used as a preprocessing step to mitigate the curse of dimensionality inherent in high-resolution spectral data [118,119]. By projecting the original spectral data onto a reduced set of principal components ordered by their explained variance, the PCA algorithm effectively reduces inter-band collinearity while preserving the dominant spectral variance with substantially fewer variables, thereby facilitating subsequent classification or regression modeling [118,119]. However, it may lose information by prioritizing features with the highest variation.

Collectively, these ML algorithms offer distinct trade-offs in HSI food quality detection. Linear methods, such as PLSR and LDA, are computationally efficient and highly interpretable but fail to capture nonlinear spectral relationships. In contrast, kernel-based methods, such as SVC, SVR, LSSVM, and LSSVR, achieve good performance in small-sample nonlinear modeling, yet their training complexity scales poorly with data size, and LSSVM and LSSVR lose sparsity, increasing prediction costs. Ensemble learners, such as RF and XGBoost, resist overfitting, provide feature importance rankings, and tolerate outliers, though they may be outperformed by well-regularized linear models when the sample size is extremely limited relative to the feature dimension. KNN requires no training and is intuitive, but its prediction time increases linearly with sample size and it suffers from the curse of dimensionality. NB is computationally fast, yet its conditional independence assumption is usually violated in raw HSI data. ELM trains fast and avoids local optima, but its random initialization causes performance instability. PCA effectively reduces inter-band collinearity while preserving dominant variance, and has been widely employed as a preprocessing step prior to modeling. As discussed below, the optimal choice or combination of these methods should be guided by datasets’ scale, mission type (classification or regression), and accuracy requirements.

### 3.2. Typical DL Algorithms

**i. Convolutional Neural Network (CNN)**. CNNs are a class of feedforward neural networks designed for grid-structured data, such as images. They employ convolutional kernels that slide over the input to extract local features, while weight sharing and local connectivity drastically reduce the number of trainable parameters [120,121]. Pooling layers perform downsampling, which enhances translation invariance and enlarges the receptive field for subsequent layers. In HSI food quality assessment, the architecture of CNNs can be tailored along different dimensions of the data cube to suit specific analytical tasks. In brief, 1D CNNs operate along the spectral axis, extracting deep features from reflectance profiles to enable prediction of internal chemical compositions [122,123]. Two-dimensional CNNs process the spatial dimensions of HSI images, capturing spatial surface features for food quality inspection [124]. In contrast, 3D CNNs combine spectral and spatial information via 3D convolutions, offering a synergistic representation that is particularly advantageous for detecting internal damage [125]. However, they require large labeled datasets to prevent overfitting, and deeper architectures might overfit if training data are scarce.

**ii. Multilayer Perceptron (MLP).** MLP is composed of an input layer, multiple fully connected hidden layers, and an output layer stacked sequentially, where each neuron in one layer connects to all neurons in the next. The network parameters are trained using backpropagation with nonlinear activation functions (e.g., ReLU and Sigmoid) to model complex relationships between spectral inputs and quality attributes. Generally, MLP offers a straightforward and adaptable architecture for modeling nonlinear relationships in spectral data. However, it is less effective at capturing local spectral–spatial features and is prone to overfitting with high-dimensional HSI data when training samples are limited. In HSI food quality detection, MLPs have been employed as baseline models for classification and nonlinear regression [125,126].

**iii. Efficient Channel Attention (ECA)**. ECA is a lightweight attention module that efficiently models local cross-channel interactions through 1D convolution. It first applies global average pooling to the input feature map to obtain a channel-wise global descriptor, then employs 1D convolution with an adaptively determined kernel size to capture local dependencies among adjacent channels. The resulting attention weights are subsequently multiplied element-wise with the original feature map, thereby recalibrating the channel-wise feature responses. This makes ECA attractive for hyperspectral feature maps, since it enhances informative spectral bands while suppressing irrelevant ones with negligible parameter overhead [127]. Nevertheless, its effectiveness depends on the representational quality of the backbone CNN and may be limited in small-sample regimes. In HSI food quality assessment, ECA is typically embedded as a plug-and-play module into the CNN backbones to enhance the importance of informative spectral bands [128,129].

**iv. Generative Adversarial Network (GAN)**. GAN is a class of deep generative models comprising two competing neural networks, a generator and a discriminator, both of which are trained through an adversarial process. The generator attempts to synthesize realistic samples from a latent space, while the discriminator learns to classify whether a sample is real or generated. This competitive training drives the generator to progressively learn the underlying distribution of the training data. In general, GANs effectively mitigate data scarcity through synthetic augmentation, improving model performance. In HSI food quality analysis, GANs have been adopted for generating synthetic spectral samples to augment limited datasets, detecting anomalies in HSI images, and enhancing their resolution [130,131,132].

**v. Variational Autoencoder (VAE)**. VAE is a generative model that extends the conventional autoencoder by incorporating probabilistic modeling into the latent space. VAE learns to represent each input as a distribution over the latent space, and is trained by jointly minimizing reconstruction error and KL divergence to a standard normal prior. This results in a continuous and regularized latent representation, which enables smooth interpolation between samples [133]. In HSI food quality studies, VAE has been used for nonlinear spectral dimensionality reduction and visualization, enabling intuitive display of food category or quality-grade distributions in a low-dimensional latent space [134,135], but its performance is sensitive to the dataset size.

**vi. Graph Convolutional Network (GCN)**. GCN is a DL method designed for graph-structured data. It operates through a neighborhood aggregation mechanism, where each node’s features are combined with those of its neighbors via a learnable transformation, enabling layer-wise propagation and update of node representations based on the graph topology. This architecture allows the GCN to effectively handle irregular data in non-Euclidean domains and capture structural dependencies between nodes [136]. In HSI applications, the data cube is typically converted into a graph by treating pixels as nodes, with edges defined by spatial adjacency or spectral similarity. Leveraging these properties, the GCN offers distinct advantages in the fine-grained classification of food samples and is well suited to irregularly shaped food samples [137,138]. However, the computational cost can be substantial for large-scale food quality assessment tasks.

**vii. Graph Attention Network (GAT)**. GAT extends GCNs by incorporating a self-attention mechanism that dynamically weights neighboring nodes during aggregation based on their feature similarity. GAT adaptively assigns higher importance to spectrally similar or spatially adjacent neighbors, which is particularly beneficial for HSI food quality analysis, where the relevance of neighboring pixels varies significantly with their spectral signatures. Recent studies have demonstrated that this attention mechanism enables GAT to achieve high-precision classification by focusing on discriminative local features [139,140]. Moreover, GAT demonstrates a notable flexibility in food adulteration detection and quality grading when spectral features vary markedly across adjacent spatial regions [141]. However, the attention mechanism introduces extra computational cost, and training GAT requires careful hyperparameter tuning for stable convergence.

**viii. Long Short-Term Memory (LSTM)**. LSTM networks are a variant of Recurrent Neural Networks (RNNs), designed to mitigate the vanishing gradient problem encountered in traditional RNNs when processing long sequential data. By introducing a cell state and three gating units (i.e., input, forget, and output gates), LSTMs dynamically regulate the flow of information across time steps, enabling them to retain or discard historical information and capture long-term dependencies within sequences. In HSI food quality studies, LSTM treats the ordered sequence of spectral bands in HSI data as sequential inputs and leverages its intrinsic sequence modeling capability to capture correlations between adjacent spectral bands [142]. Additionally, LSTM is often combined with CNN to form hybrid architectures. In such architectures, CNNs extract spatial features from hyperspectral images, while LSTMs capture spectral dependencies along the wavelength dimension, making the overall framework well suited for complex food classification tasks [143].

**ix. Gated Recurrent Unit (GRU).** GRU is a computationally efficient gated architecture derived from the LSTM. By modulating temporal information flow through an update gate and a reset gate, GRU effectively mitigates the vanishing gradient problem inherent in conventional Recurrent Neural Networks (RNNs) when processing long sequential data. The architecture omits the separate memory cell, yielding a substantially reduced parameter count compared to LSTM, which accelerates training convergence and renders it suitable for computationally constrained or online inference scenarios. In HSI food quality detection, GRU is often integrated with CNNs to form hybrid architectures [144]. In this strategy, the CNN first extracts shallow spatial and spectral features from the HSI data; the resulting feature maps are then unfolded along the wavelength axis into sequential representations, which are subsequently fed into the GRU to capture long-range spectral dependencies across adjacent bands. This synergistic framework enables precise qualitative classification and quantitative regression of food attributes. However, its performance can degrade when spectral bands exhibit complex and multi-scale dependencies.

**x. Stacked Autoencoder (SAE)**. SAE is built by stacking multiple autoencoder layers. It employs a layer-wise unsupervised pre-training strategy, in which each autoencoder is trained to minimize reconstruction error to learn a compressed representation of the input. The trained encoder layers are then stacked to initialize a deep network, which is then fine-tuned in a supervised manner using backpropagation to optimize the classification or regression performance. This two-stage learning strategy enables the SAE algorithm to extract low-dimensional spectral features from high-dimensional HSI data [145,146]. However, the two-stage training process is computationally intensive, and the quality of extracted features depends heavily on the choice of network architecture and pre-training hyperparameters.

To enhance prediction performance in HSI food quality detection, hybrid models have been explored to overcome the limitations of single DL models. Spatial–spectral feature complementarity strategies, realized through hybrid models such as CNN-LSTM and CNN-GRU, have been adopted. In this framework, the CNN extracts local spatial and shallow spectral features from HSI images, whereas the LSTM or GRU capture sequential spectral dependencies across the wavelength dimension [85,144]. As shown below, these combined methods have proven effective in quality evaluation of freeze–thawed chicken breast and surimi gel. In addition, researchers have combined attention mechanisms with gated units, such as the CNN-DotGRU–SelfAttention model, which leverages a dot-product GRU and a self-attention mechanism, to strengthen the generalization of models across various species [147]. Furthermore, the integration of data transformation and multi-task learning, such as Stacking SDAE-PLSR-RR, has been studied to predict multiple quality indicators of apples across different processing conditions [94]. This design integrates unsupervised deep feature extraction and supervised linear regression fine-tuning, thereby achieving a balance between deep feature richness and linear model interpretability. Table 1 shows recently reported hybrid DL models used in HSI food quality detection.

## 4. Typical Applications of the ML and DL Algorithms in Hyperspectral Imaging Food Quality and Safety Detection

### 4.1. Food Quality Assessment

#### 4.1.1. Fruit and Vegetable Quality

**Figure 4:**
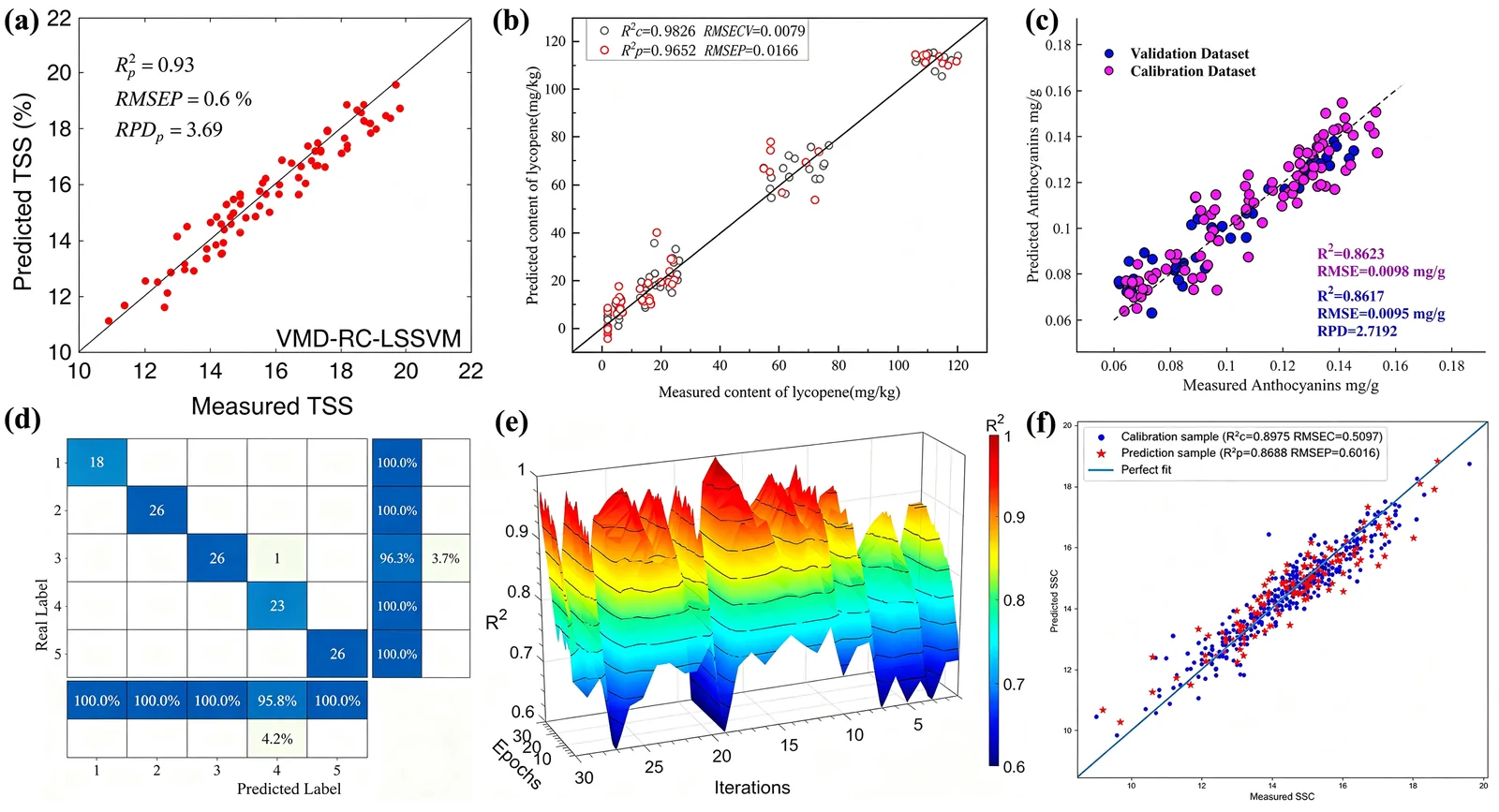
(**a**) Predicted and measured total soluble solids (TSS) for the VMD-RC-LSSVM model [106]. (**b**) Predicted and measured lycopene content values for the SVR model [150]. (**c**) Predicted and measured anthocyanin content in purple-leaf lettuce for the UVE-SNV-CARS-DBO-ELM model [70]. (**d**) Confusion matrix of POA-CDGSA-Net on the test set for chilling injury identification [147]. (**e**) R^2^ value of the MA-CNN model under five-fold cross-validation [148]. (**f**) Predicted and measured SSC based on SDAE-PLSR-RR [94].

HSI combined with ML and DL algorithms has gained marked attention in the quality evaluation of fruits and vegetables. It has been shown that feature selection can effectively improve model efficiency and accuracy. For instance, in the detection of SSC in apples, the predictive model established after extracting characteristic wavelengths using CARS and CNN algorithms achieved higher prediction accuracy than the full-spectrum model [102,148]. In brief, CARS eliminates redundant variables via adaptive reweighted sampling, and the resulting wavelengths are then fed into CNNs to further extract nonlinear spectral features, collectively optimizing the input information for the regression model. Meanwhile, in the detection of SSC in grapes, the LSSVM model, coupled with characteristic wavelengths selected by the VMD-RC algorithm, achieved an RPD of 3.69 (Figure 4a) [106]. Specifically, VMD decomposes the raw spectra into intrinsic mode functions, and RC identifies the most informative frequencies related to SSC. Moreover, nonlinear models, such as SVR, DBO, SABO, and WOA, generally perform well when dealing with complex biochemical indicators, such as lycopene in tomato (Figure 4b) [150] and anthocyanins in purple-leaf lettuce (Figure 4c) [70]. Generally, these nonlinear models outperform linear models (such as PLSR) in predicting such complex biochemical indicators, as they are more capable of capturing the complex and nonlinear relationships between spectral signatures and target compound concentrations. Interestingly, emerging spectroscopic techniques, including terahertz time-domain spectroscopy (THz-TDS), have offered alternative strategies for specialized detection, such as the nutritional status of phosphorus in lettuce [151].

Notably, DL has ushered in a paradigm shift in HSI food quality detection, moving the field from hand-crafted feature selection toward end-to-end automatic feature learning. Typically, in online detection of SSC in strawberries, the 1D-CNN-LSTM model, which directly utilizes raw spectral data, outperformed the multi-calibration CARS-PLSR model [36]. This performance advantage may suggest that the 1D-CNN-LSTM model is capable of automatically learning spectral features from raw data and effectively captures complex and nonlinear variations that are often missed by traditional two-step approaches relying on manual feature engineering. Similarly, the hybrid deep network of CNN-DotGRU-SelfAttention (CDGSA-Net) achieved a classification accuracy of 99.17% for cross-variety identification of early cold injury in kiwifruit (Figure 4d) [147]. This network enhances CNN-extracted features by introducing a dual-channel dot-product attention mechanism and integrates gated recurrent units (GRU) and a self-attention (SA) mechanism to model sequential dependencies and internal feature correlations, effectively capturing both common and variety-specific spectral signatures of chilling injury. Moreovers, in the detection of SSC in apples, the multi-attention convolutional neural network (MA-CNN) (Figure 4e) [148] and the stacked SDAE-PLSR-RR model that combines stacked denoising autoencoders with PLSR and ridge regression (RR) (Figure 4f) [94] both surpass the performance limits of traditional methods. Meanwhile, DL has proven strong robustness in a range of complex detection tasks, such as early bruise detection [152], selenium content prediction [28], and cold injury and chilling identification [37,153]. These successes demonstrate that DL algorithms can accommodate spectral variations arising from differences in varieties and growing conditions in practical applications.

Recent efforts have increasingly focused on model lightweighting, multi-task learning, and interpretability enhancement, aiming to accelerate the deployment of HSI techniques from lab studies to practical applications. On the one hand, efficient training strategies, such as Bayesian optimization algorithms (BOAs), have been explored to automatically search for network hyperparameters [148]. In addition, multi-objective feature selection algorithms such as distinguished index-modified CARS (DI-CARS) [154] have been explored to select characteristic wavelengths associated with multiple quality indicators. On the other hand, efforts have been devoted to improving model interpretability. For example, the Shapley additive explanation (SHAP) has been employed to analyze the importance of features to explain the model’s classification, where each feature’s contribution is quantified to the final prediction, thereby providing valuable insights into the underlying chemical and physical origins in the food samples [37].

#### 4.1.2. Meat Quality

**Figure 5:**
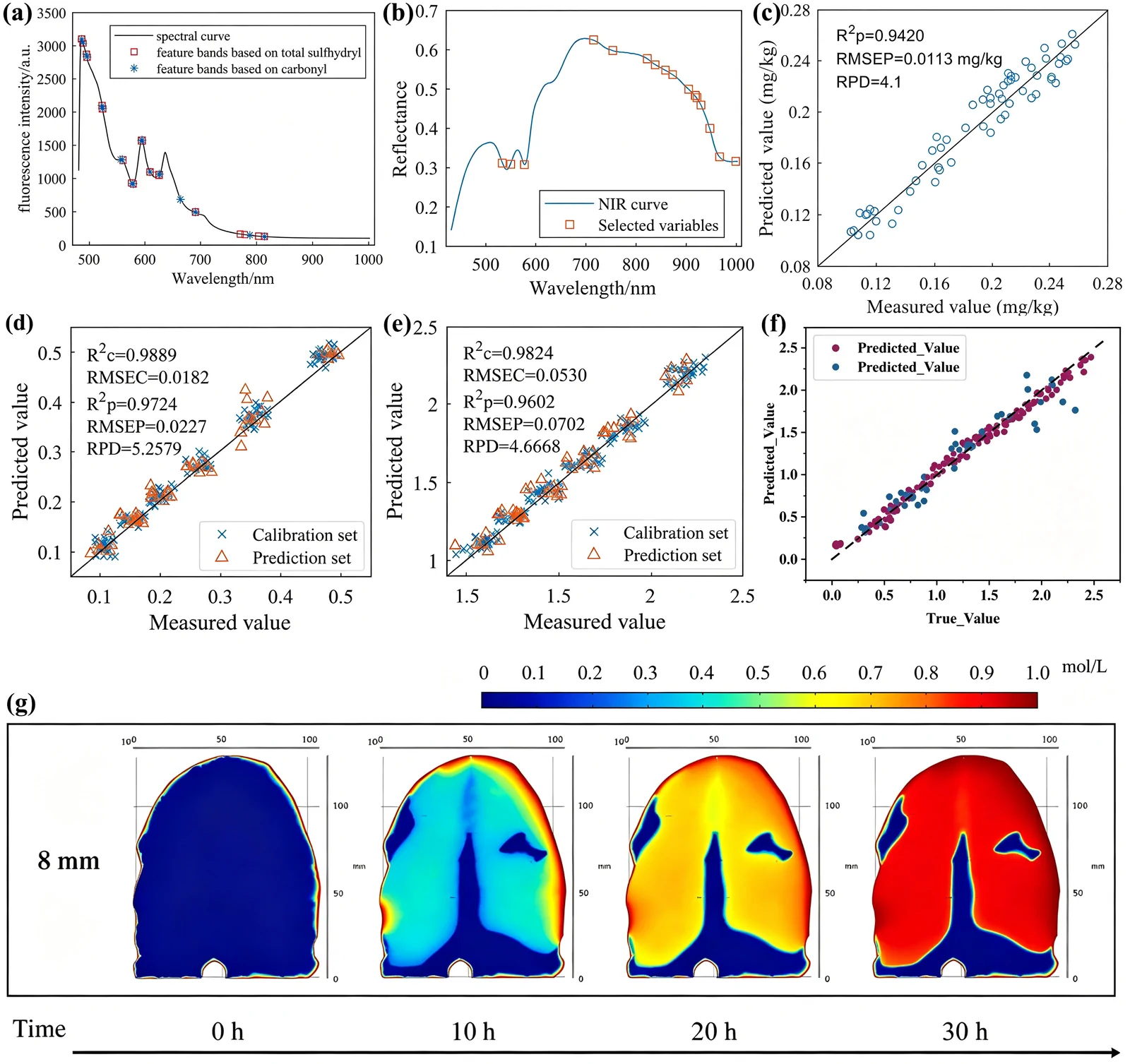
Feature wavelengths of (**a**) protein oxidation [155] and (**b**) moisture [156] in frozen–thawed pork extracted by MI-VIF methods. (**c**) Predicted and measured TBARS contents in the frozen pork [157]. Predicted and measured (**d**) TABRS and (**e**) carbonyl content in the frozen–thawed pork [160]. (**f**) Prediction scatter plot of the freeze–thaw cycled chicken breast [85]. (**g**) Visualization of 3D diffusion behavior of sucrose in marinated beef [163].

Traditional ML methods, typically PLSR, when combined with spectral preprocessing and characteristic wavelength extraction, have reached a mature stage in the quantitative prediction of single quality indicators in meat. These methods have also been extended to visualize chemical indicator distributions. In a series of studies on frozen–thawed pork, Cheng et al. employed PLSR to predict protein oxidation (R^2^_p_ = 0.9275 [155]) and moisture content (R^2^_p_ = 0.9533 [156]), where the Mutual Information-Variance Inflation Factor (MI-VIF) was used to extract the characteristic wavelengths (Figure 5a,b). Meanwhile, they used the Bootstrapping soft shrinkage-Gaussian process regression (BOSS-GPR) hybrid model to achieve highly accurate quantification of lipid oxidation (TBARS) with an R^2^ of 0.9726 (Figure 5c) [157], underscoring the value of advanced wavelength selection in meat quality assessment. In brief, BOSS selects feature wavelengths highly correlated with TBARS via weighted bootstrap sampling, and then GPR models the selected features, effectively capturing the complex nonlinear biochemical changes during lipid oxidation. Notably, the BOSS-GPR hybrid model exhibited superior predictive accuracy compared to the PLSR models, indicating that combining advanced wavelength selection with nonlinear regression can more effectively capture the complex biochemical changes associated with lipid oxidation [157]. In addition, Aheto et al. achieved favorable results (R^2^ = 0.899) by integrating spectral and texture information within a PLSR framework in pork [158]. In recent years, the applicability of PLSR methods has been studied across diverse meat foods and seafood products. In chicken, Khulal et al. combined the PLSR model with the Ant Colony Optimization (ACO) algorithm to predict TVB-N content, demonstrating an R^2^ of 0.7542, where ACO efficiently extracts the spectral features correlated to the TVB-N content of chicken via accumulation of information in the form of pheromone trails deposited on each wavelength [159]. In shrimp flesh, Xi et al. achieved superior predictions of TVB-N (R^2^ = 0.9431) by integrating the PLSR with IRIV feature selection [87]. In addition, Xia et al. employed PLSR to monitor the changes in gel quality, water-holding capacity, and whiteness during thermal processing of surimi, with the R^2^ exceeding 0.93 [84]. Recently, Cheng et al. employed fluorescence HSI combined with PLSR to predict pork protein oxidation [155] and lipid oxidation [157], suggesting the complementary advantages of distinct imaging techniques. Collectively, these studies show that finely optimized traditional ML models can achieve both high accuracy and strong robustness for moderate sample sizes and single-metric regression.

To mitigate the inefficiency of building separate models for each quality indicator, DL algorithms have been studied to enable multi-task learning and unified feature extraction within a single framework. Typically, Cheng et al. have reported a multi-task CNN that simultaneously predicts lipid oxidation (R^2^ = 0.9724) and protein oxidation (R^2^ = 0.9602) in frozen–thawed pork (Figure 5d,e), which outperformed single-task CNN and PLSR [160]. This multi-task approach simultaneously improves prediction accuracy and enhances model efficiency by sharing features across related tasks. In addition, Yao et al. converted 1D spectra into 2D images using Gramian angular difference fields (GADF) and further used a multi-task CNN (GADF-MTCNN) to simultaneously detect the TBARS (R^2^ = 0.9458) and carbonyl content (R^2^ = 0.9545) within frozen–thawed chicken [149]. For extracting complex nonlinear spectral features, Li et al. have constructed a CNN-LSTM hybrid model incorporating an attention mechanism, achieving high prediction accuracy for freeze–thaw cycle chicken breast (R^2^ = 0.976) (Figure 5f) [85]. This hybrid model uses CNN layers to efficiently extract local spectral motifs, such as sharp chemical absorption peaks, and employs LSTM to capture long-range sequential dependencies across the continuous spectral domain, collectively enabling robust feature learning even with moderate sample sizes. Notably, the CNN-LSTM hybrid model has shown improved performance in predicting the surimi gel quality (R^2^ = 0.9431), compared to the results obtained by single CNN or LSTM models [87] and the CNN-GRU (Gated Recurrent Unit) hybrid model (R^2^ = 0.9224) [144]. To achieve high accuracy in freshness discrimination, fluorescent HSI techniques combined with the Hybrid Attention Fusion Network (HFA-Net) [161] and light scattering imaging combined with adaptive boosting orthogonal linear discriminant analysis (AdaBoost-OLDA) [162] were proposed. Specifically, HFA-Net incorporates early fusion with an attention mechanism into a late fusion architecture, simultaneously capturing cross-modal correlations between HSI spectra and electronic nose signals. In contrast, AdaBoost-OLDA iteratively constructs a cascade of weak classifiers by re-sampling and constructing calibration sets, and then integrates them via weighted majority voting to achieve high-precision classification of subtle texture variations in light scattering images.

Due to the pixel-wise spectral information provided by HSI, predictive models can be applied to each pixel, enabling spatial distribution maps of the predicted quality indicators. Recently, optimal models have been explored to visualize moisture [62], lipid oxidation [157], protein oxidation [155], and salt distribution [158] in pork, providing an intuitive display of quality heterogeneity induced by curing processes and freeze–thaw cycles. This visualization capability is an important advantage of HSI, facilitating the interpretation of model predictions at a spatial level and allowing the identification of localized quality variations that are invisible to conventional analytical methods. In addition, HSI visualization has been further combined with finite element analysis (FEA) to study sucrose diffusion in marinated beef (Figure 5g) [163]. Meanwhile, HSI-FEA hybrid models were demonstrated to investigate the effect of the Gaidao process on the quality of marinated beef [164]. Separately, Tian et al. integrated the HSI with CLSM and GC-IMS to assess dried pork quality by detecting chemical, structural, and volatile changes, showing the synergistic application of HSI techniques with various characterization methods to enhance data mining and interpretation [62].

#### 4.1.3. Egg Quality

**Figure 6:**
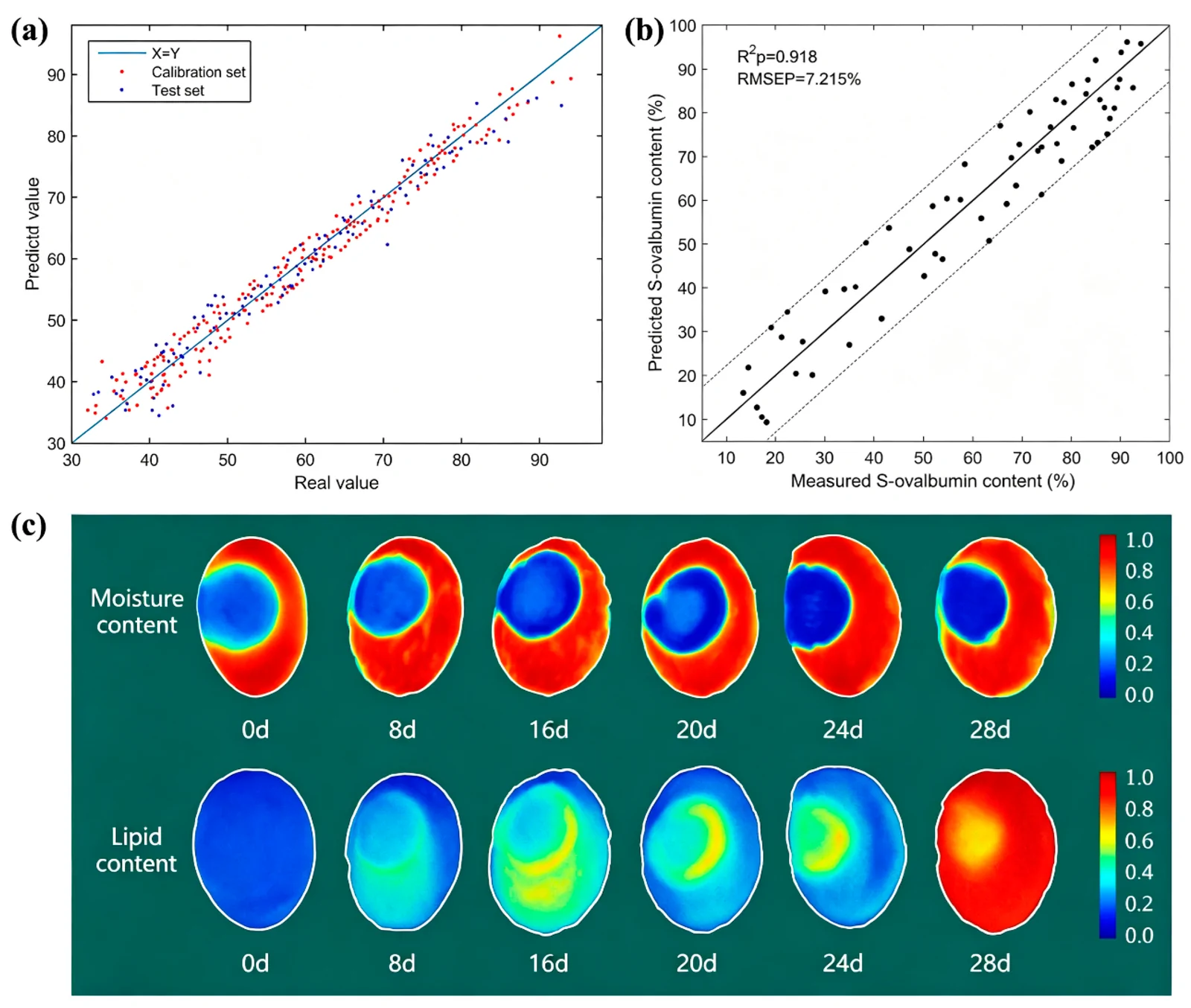
(**a**) Predicted and measured Haugh unit content of the HHO-BOSS-SVR model in eggs [166]. (**b**) Predicted and measured S-ovalbumin content of the LSSVM models in eggs [167]. (**c**) Visualization of the distribution of moisture and lipid content in eggs [165].

Recently, significant advancements have been achieved in the quantitative prediction of egg internal components and product quality grading through the combined application of ML, DL, and HSI techniques. In quantitative analyses, PLSR models were constructed to predict various quality indicators, such as moisture, lipid, and salt content, during salted duck egg processing [165]. Following preprocessing with SG smoothing, SNV, and MSC, and feature wavelength selection via CARS and UVE, the R^2^ for protein moisture, yolk moisture, yolk lipid, and protein salt content reached 0.8200, 0.8969, 0.8709, and 0.9239, respectively [165]. In egg freshness detection, a Genetic Algorithm (GA)-optimized SVM combined with IRIV achieved a classification accuracy of 97.87% for freshness grading [98]. In that study, IRIV selects spectral features, and GA searches for the optimal penalty coefficient and kernel function width of SVM, together yielding a robust prediction model with reduced overfitting. Separately, the Harris Hawks Optimization SVR (HHO-SVR) model achieved an R^2^ of 0.9523 and an RMSEP of 3.0423 for predicting Haugh unit values (Figure 6a) [166]. Similarly, HHO is used to optimize the penalty factor and kernel parameter of SVR via a ‘surprise pounce’ mechanism combined with Lévy flight, enabling rapid convergence and high accuracy. In addition, for freshness indicator S-ovalbumin content detection, an LSSVM model was established, yielding a prediction R^2^ of 0.918 and an RMSEP of 7.215% (Figure 6b) [167].

Notably, multi-source data fusion strategies coupled with advanced image information mining have enhanced the comprehensiveness and accuracy of detection performance. Typically, Ren et al. have developed a data fusion strategy that integrated electronic nose signals with reflectance hyperspectral and transmittance hyperspectral data, which were then jointly modeled using PCA-LDA [168]. This multi-sensor fusion approach enhanced the classification accuracy of preserved eggs at different maturity stages, elevating it from 88.89% to 95.56% [168]. This improvement suggests that volatile organic compounds detected by the electronic nose provide complementary information to the spectral data. In defect detection, Yao et al. conducted a multi-task investigation using transmission HSI [169]. For egg freshness prediction, they extracted 16 characteristic wavelengths via the IRIV algorithm and developed an XGBoost regression model, which yielded an R^2^_p_ of 0.91 and an RMSEP of 4.64. For scattered yolk detection, they employed an image processing pipeline at the 570 nm band, where both the signal-to-noise ratio and contrast between yolk and other areas are high, to extract yolk morphological features, achieving a classification accuracy of 97.33%. Meanwhile, for eggshell crack detection, they enhanced crack features using a Laplacian of Gaussian (LoG) operator and applied an XGBoost model, achieving a recognition accuracy of 93.33%. These results show that the HSI technique can simultaneously conduct both quantitative and qualitative quality assessment within a unified analytical pipeline, significantly improving the efficiency of egg quality control. In addition, visualization distribution maps translate spectral data into intuitive spatial representations, revealing the dynamic changes in water and lipid distribution during salted duck egg processing (Figure 6c) [165], as well as the localized accumulation of S-ovalbumin during egg storage [167]. Collectively, these studies have shown that HSI techniques combined with ML and DL algorithms enable simultaneous quantitative analysis of internal egg components, freshness grading, and external defect detection, establishing a rapid and non-destructive evaluation strategy for eggs.

#### 4.1.4. Tea Quality

In recent years, HSI techniques have been extensively applied in conjunction with ML and DL algorithms for tea quality assessment, covering diverse product forms including matcha, green tea, black tea, and oolong tea. For qualitative classification, tea attributes, such as grade [170], variety [171], and moldiness [172], have been evaluated by combining ML algorithms, such as PLS, RF, SVM, and LSSVR. Notably, the VISSA-ABC-SVM models obtained an impressive accuracy of 97.4% in Tieguanyin tea grade classification [173]. This high accuracy was achieved by using VISSA to iteratively optimize variables via weighted binary matrix sampling, thereby selecting the most informative spectral bands, while the ABC algorithm efficiently searched for the optimal penalty factor and kernel function width of the SVM, collectively enhancing the model’s classification performance and generalization. For quantitative prediction, PLS and LSSVR models have been employed to assess important chemical constituents such as tea polyphenols, crude fiber, caffeine, free amino acids, and chlorophyll [171,174], and ANN models to estimate the particle size distribution [175] and sensory quality scores [176]. In quantitative prediction, Tian et al. have reported predicting the astringency and aftertaste in differently processed green teas by combining hyperspectral appearance information with PLSDA and SVR models, yielding an R^2^ exceeding 0.90 [177]. Separately, Li et al. used microscope HSI combined with interval random frog (iRF)-SPA-PLS hybrid models to assess the matcha color physicochemical indicators (L*, a*, b*) and chlorophyll content [61]. In that study, the iRF algorithm selected informative spectral intervals from the full spectrum through random frog sampling, while the SPA further eliminated collinear and redundant variables within these intervals, yielding the PLS model with high prediction accuracy for each color-related indicator. To improve model performance, various data fusion strategies have been explored, such as combining spectral features with image texture features [175] and combining HSI with olfactory visualization systems [178]. In parallel, feature selection algorithms, such as CARS, iRF, SPA, iRF-SPA, and BOSS, have been further explored to reduce data redundancy and model complexity, and improve predictive stability [61,174]. Among these, BOSS generally performs well in quantifying multiple chemical constituents simultaneously, as its model population analysis and soft shrinkage strategy provide more robust variable importance assessment by leveraging information from a large population of submodels rather than a single model, thereby mitigating the adverse effects of collinearity among spectral bands.

In contrast, DL algorithms exhibit distinct advantages in exploiting spatial information within HSI data, due to their superior feature extraction capabilities and strong nonlinear fitting capacity. Recently, CNNs have been explored for tea quality classification. For example, Ding et al. applied a ResNet-50 to process PCA-processed data, leveraging its hierarchical feature extraction capability to capture discriminative spatial patterns [170]. Specifically, PCA extracted the first three principal component images (PC1–PC3) from 461-spectral-band HSI images, which were then fed into ResNet-50. The resulting model achieved 92.3% accuracy across 13 different quality grades, notably exceeding that of traditional ML baselines, showing the potential of DL for high-precision tea grading. Meanwhile, the 1D-ResNet18 model has also been employed for tea cultivar identification, achieving 99.6% accuracy in classifying 14 tea cultivar plants [171]. The near-perfect classification accuracy underscores CNNs’ exceptional ability to identify cultivar-specific spectral features, benefiting from the model’s capacity to learn hierarchical representations that gradually encode both broad spectral trends and detailed discriminative features.

### 4.2. Moisture Content Detection

**Figure 7:**
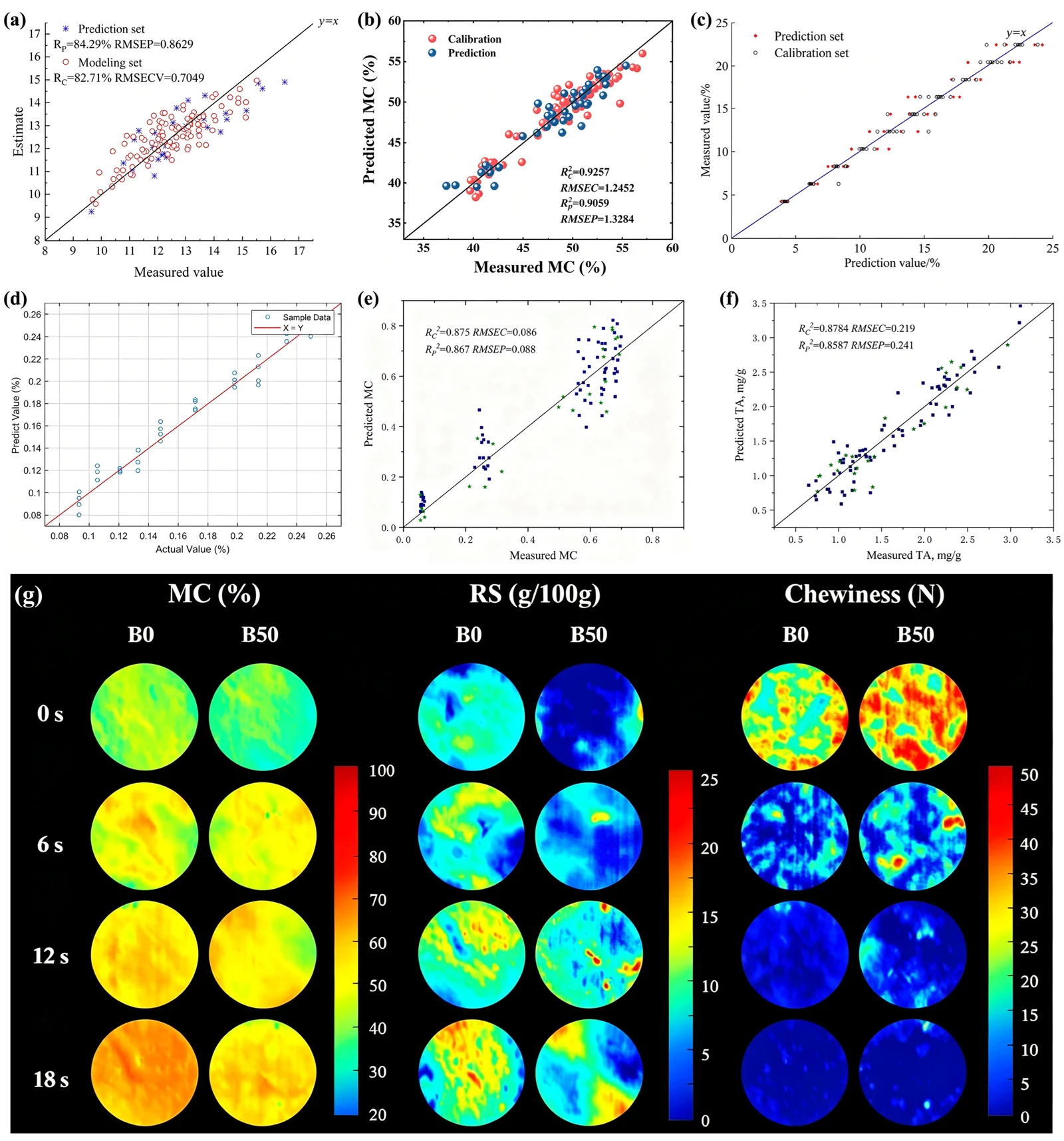
(**a**) MCUVE-CARS-PLS model for predicting water content in lettuce canopies [179]. (**b**) SNV-PLSR model for predicting moisture content in bread during oral processing [181]. (**c**) SAGA-SVR model for predicting moisture content in rice seeds [182]. (**d**) MSLPP-ESMA-SVR model for predicting moisture content in rice (with husk) [184]. Measured and predicted values of (**e**) moisture content and (**f**) total anthocyanins (TA) contents of dried purple sweet potatoes [185]. (**g**) Distribution map of moisture content, reducing sugars, and chewiness of two types of bread at different stages of oral processing [180].

ML algorithms have markedly advanced the application of HSI techniques for moisture content detection in various foods, such as cereal grains (e.g., wheat and rice), fruits and vegetables (e.g., lettuce, purple sweet potatoes, and mangoes), and processed products (e.g., bread and vinegar starters). Various preprocessing methods, such as MSC, SNV, SG smoothing, and FD, were used to process raw HSI data to eliminate noise and baseline drift. Subsequently, characteristic wavelengths were extracted using CARS, Monte Carlo uninformative variable elimination (MCUVE), modified supervised locality preserving projections (MSLPP), SPA, PCA, and WT. Finally, quantitative prediction models were established by combining ML models such as PLSR, SVR, and RF. Typically, Wu et al. achieved an R^2^ of 0.713 for wheat leaf moisture detection using the MSC-CARS-SVR model [92]. Zhao et al. applied FD preprocessing with MCUVE-CARS feature selection, yielding a PLS prediction correlation (r) of 0.8467 for moisture content in lettuce canopies under outdoor conditions (Figure 7a) [179]. Zhou et al. extracted characteristic wavelengths using WT-PLSR, yielding an R^2^ of 0.8307 for detecting moisture content in lettuce leaves [95]. Tian et al. have established a PLSR model for dough moisture content during bread processing, attaining an R^2^ of 0.8898 [180]. Subsequent work further enhanced predictive performance by using SNV preprocessing of the HSI data, which elevated the R^2^ value to 0.9059 (Figure 7b) [181]. Lu et al. reported an R^2^ of 0.9318 for detecting rice seed moisture using a simulated annealing Genetic Algorithm (SAGA)-SVR (Figure 7c) [182], while Sun et al., using BCC-LS-SVR-SPA, achieved a prediction R^2^ of 0.989 for rice moisture [183]. Collectively, these findings suggest that integrating HSI techniques with ML algorithms offers a robust platform for rapid, non-destructive, and quantitative detection of moisture across diverse food matrices.

Notably, algorithm optimization has proven critical to enhancing model performance. Zhong et al. combined the MSLPP feature extraction algorithm with an ESMA-optimized SVR model, achieving an R^2^ of 0.9755 for husk rice moisture prediction (Figure 7d) [184]. Bai et al. employed laser backscattering imaging with a Gaussian amplitude function for feature extraction, reaching an R^2^ of 0.9247 for mango slice moisture content detection [186]. Alternatively, Tian et al. constructed a PLSR model by integrating the spectral data with GLCM texture features, obtaining an R^2^ of 0.867 and 0.859 for monitoring moisture and anthocyanin levels, respectively (Figure 7e,f) [185]. In addition, visualization has been leveraged to monitor both static and dynamic food processes. Zhu et al. employed HSI to map the spatial distribution of moisture and total acidity during vinegar solid-state fermentation, enabling an intuitive assessment of fermentation uniformity [187]. In addition, Tian et al. have visualized the spatial distribution of moisture content, reducing sugars, hardness, and chewiness within bread during oral processing, offering insights into the moisture and compositional changes in foods during mastication (Figure 7g) [180,181]. Collectively, HSI combined with ML algorithms offers a reliable technical pathway for rapid and nondestructive moisture content prediction, which may facilitate online monitoring and quality control in smart agriculture and the food industry.

### 4.3. Varieties and Origin Identification

**Figure 8:**
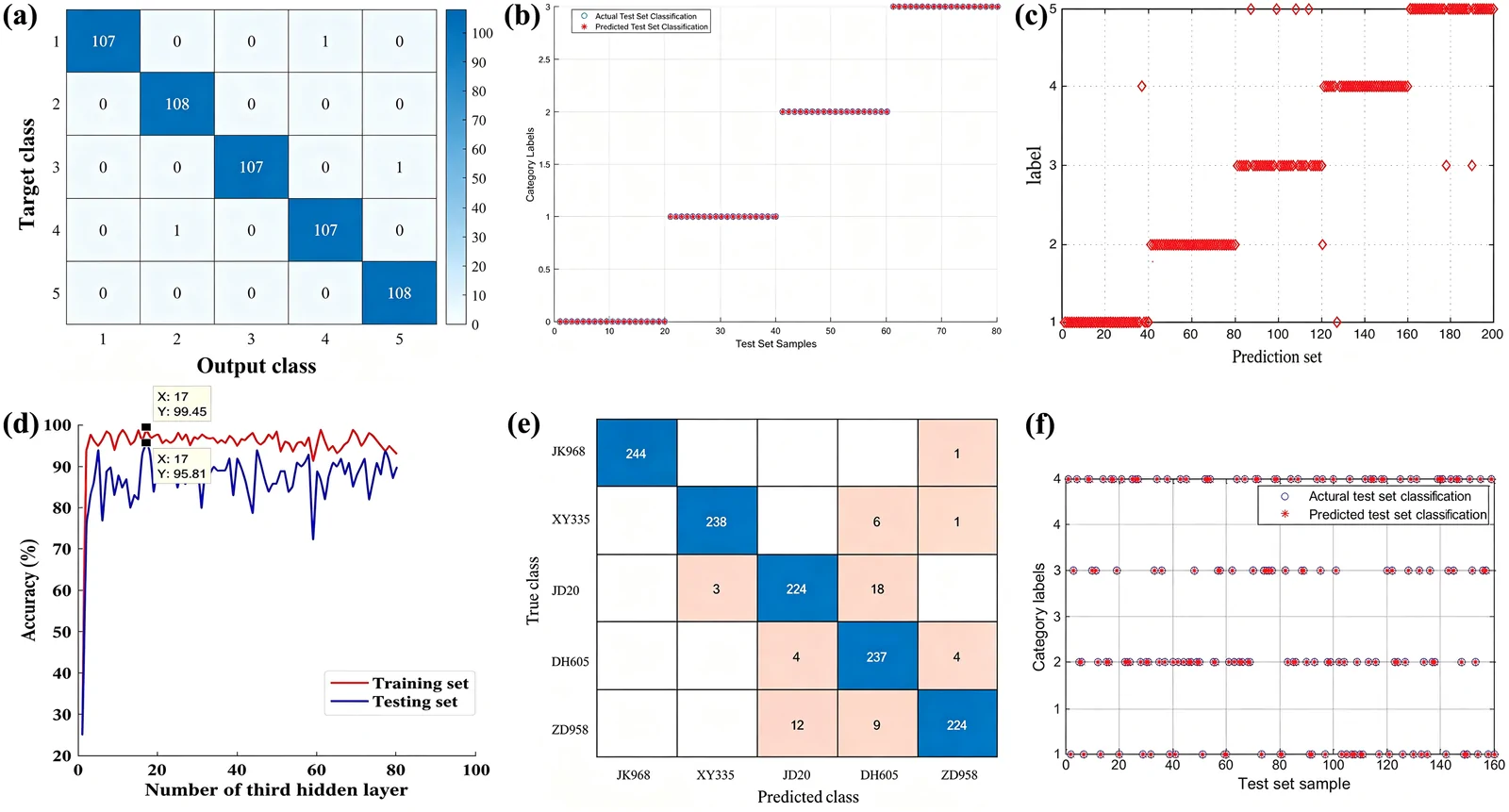
(**a**) Confusion matrix of AFSA-SVM models for identifying rice seed varieties [189]. Classification of tea varieties based on (**b**) fluorescence HSI and ABC-SVM model [27] and (**c**) VISSA-FA-SVM models [191]. (**d**) Classification of maize seed varieties based on SSAE-CS-SVM models [97]. (**e**) Confusion matrix of CNN-LSTM models for detecting five maize varieties [195]. (**f**) Classification of grape varieties based on the EEMD-DWT-CARS-SPA models [76].

ML and DL algorithms have markedly advanced the application of HSI to crop variety identification. Early studies in this area predominantly centered on integrating ML models with manual feature engineering. For instance, Sun et al. combined SVM with spectral, morphological, and texture features to determine rice origins, achieving an accuracy of 91.67% [188]. In a subsequent study, Sun et al. employed the BOSS algorithm for feature selection and integrated information fusion with AFSA-optimized SVM, improving the identification accuracy for rice seed varieties to 99.44% (Figure 8a) [189]. The enhanced prediction accuracy results from the combined strengths of BOSS in feature selection and AFSA in global parameter optimization. Specifically, BOSS iteratively selects optimal spectral and image features via weighted bootstrap sampling and model population analysis, while AFSA optimizes the SVM penalty factor and kernel range, together yielding a robust classification model. In tea variety identification, Ge et al. combined the BOSS algorithm with LightGBM, attaining 97.33% accuracy for oolong tea varieties discrimination [190], while Ahmad et al. used CARS- and ABC-optimized SVM to achieve a 100% recognition rate for oolong tea varieties (Figure 8b) [27]. Notably, the perfect accuracy of the ABC-SVM model suggests that fluorescence HSI data, when coupled with the swarm intelligence optimization algorithm, can effectively capture subtle chemical differences among tea cultivars. Alternatively, Sun et al. employed a variable iterative space shrinkage approach (VISSA) and firefly algorithm (FA)-optimized SVM to achieve a 96% recognition rate for green tea varieties (Figure 8c) [191]. In detail, VISSA iteratively optimizes the variables via weighted binary matrix sampling to select key feature wavelengths, while the FA optimizes the parameters of SVM, resulting in a highly accurate classification model. In black bean variety classification, Sun et al. built PLS-DA models based on the combination of spectral and image features, achieving an accuracy of 98.33% [192]. Beyond the crops discussed above, the SVM classifier coupled with feature selection methods, such as CARS, PCA, stacked sparse autoencoder (SSAE), IRIV, and VISSA, has proven broadly applicable across diverse foods, including Lycium barbarum [189], corn [97], red jujube [193], and apples [194], with accuracies falling within the 85–98% range. These findings indicate that effective feature extraction, strategic feature fusion, and rigorous parameter optimization are critical to enhancing the performance of ML models for food variety classification.

The introduction of DL algorithms has advanced the accuracy of species identification and enhanced the generalization ability of models. Conventional chemometric methods, such as PLS-DA and SVM, often struggle with the high-dimensionality and nonlinearity inherent in hyperspectral data. To address this, Fu et al. employed the SSAE algorithm to extract deep features from near-infrared hyperspectral data, which were then integrated with a cuckoo-search-optimized SVM (CS-SVM), achieving an accuracy of 95.81% for corn seed variety discrimination (Figure 8d) [97]. The high-performance shows that deep unsupervised feature extraction can effectively capture nonlinear hidden features that linear dimensionality reduction methods always fail to reveal. Alternatively, Zhu et al. built a CNN-LSTM hybrid model that directly classifies maize seed varieties, achieving 95.27% accuracy (Figure 8e), which substantially outperforms traditional chemometric models such as PLS-DA, SVM, and ELM models [195]. The enhanced performance of the CNN-LSTM hybrid model is due to CNN’s effective extraction of local spectral features and LSTM’s ability to capture long-range sequential dependencies across wavelengths. As a result, the combination of deep feature extraction with conventional classifiers seems to attest to the flexibility and robustness of DL models for complex food variety identification. In grape variety identification, Xu et al. employed a multi-step strategy that combined EEMD-DWT denoising with CARS-SPA wavelength selection and SVM modeling [76]. This boosted classification accuracy from 94.31% to 99.31% (Figure 8f). These studies show that DL models effectively extract deep nonlinear features from the HSI data while maintaining superior robustness and generalization in complex classification tasks. Collectively, the HSI techniques combined with ML and DL algorithms have shown considerable promise for food variety identification.

### 4.4. Additive and Adulteration Detection

**Figure 9:**
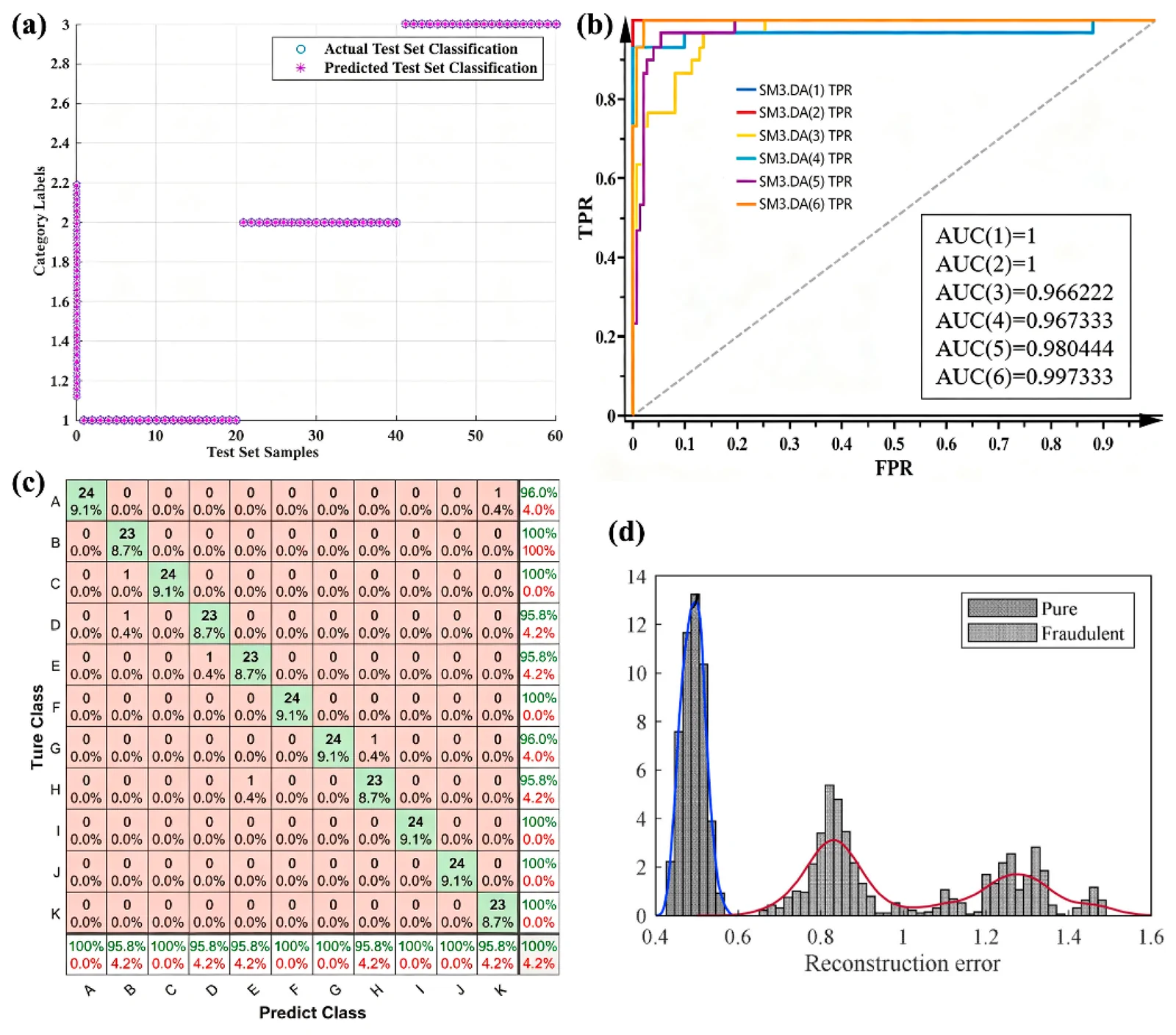
(**a**) Classification of aged sulfur-fumigated wolfberries, freshly dried wolfberries, and aged non-fumigated wolfberries based on CARS-GA-SVM models [196]. (**b**) Area under the curve values based on ICA-spectral fusion data for detecting beef adulteration with adulteration rates of 0, 10%, 20%, 30%, 30%, 40%, and 50% [199]. (**c**) Confusion matrix of VGG16-SVM models for the identification of soybean protein with mass fractions of 0, 5%, 10%, 15%, 25%, 30%, 35%, 40%, 45%, and 50% in minced chicken meat [39]. (**d**) Histograms of reconstruction errors for pure and fraudulent (with syrup concentrations of 5%, 10%, 25%, and 50%) Manuka honey of validation data [131].

ML and DL algorithms have been extensively integrated with HSI for food adulteration detection, demonstrating considerable effectiveness across diverse food matrices. At the preprocessing and feature extraction level, the combination of traditional ML with HSI has matured into well-established analytical approaches. Recently, various techniques, such as MSC [196], SNV [39], SG [197], MC [197], and continuous wavelet transform (CWT) [38,39], were used to reduce noise, baseline drift, and scattering effects. Among these, CWT offers advantages in that it converts 1D spectra into 2D spectral images, enhancing subtle spectral features [38,39]. For instance, in the detection of soybean protein adulteration in minced chicken, CWT preprocessing elevated the classification accuracy of the CNN model to 90.5%, markedly outperforming the 83.5% achieved by SG-SNV, 81.2% by SG-SNV-CWD, and the 89.1% by FD preprocessing, confirming CWT’s superior capability in extracting weak spectral signatures that are critical for distinguishing chemically similar adulterants [39]. For feature selection, a variety of feature selection algorithms have been employed, such as CARS [25,100,196,197], SPA [25,197], iRF [197], and LassoNet algorithm [198]. These methods effectively reduce spectral redundancy and enhance model efficiency. On this basis, traditional supervised learning models, such as SVM [25,38,100,196,198], PLS [197,199], LDA [25,86,199], and KNN [25,86,196,199], have delivered reliable performance in both qualitative classification and quantitative prediction of adulteration detection across diverse foods. For example, SVM models coupled with CARS showed 98.75% accuracy for sulfur-fumigated wolfberries classification (Figure 9a) [196]. In addition, LDA delivered perfect discrimination between reconstituted and pure beef, and PLS-DA hybrid models for detecting pork adulteration in beef yielded area under the curve values exceeding 0.92 (Figure 9b) [199]. Notably, traditional and DL-based one-class classifier methods have been explored for honey authenticity verification without requiring adulterated samples for model training [131]. Overall, ML algorithms retain distinct advantages in applications that depend heavily on feature selection and involve moderately sized datasets, demonstrating robust generalization and strong practical utility.

The emergence of DL has markedly advanced HSI food adulteration detection, yielding notable improvements in autonomous feature learning and the discrimination of nonlinear and complex signatures. CNNs and their variants, such as GoogLeNet [38], VGG16 [39], and transfer learning [38,39], can learn deep features from CWT-derived 2D spectrograms, circumventing the inherent subjectivity and constraints associated with manual feature engineering. As reported, VGG16 combined with SVM yielded an accuracy of 98.1% for the assessment of soy protein adulteration in chicken meat (Figure 9c) [39]. Specifically, the VGG16-SVM model was created by substituting the fully connected layer of the VGG16 network with an SVM, where features extracted by VGG16 are used to train the SVM for classification. Meanwhile, the GoogLeNet-based transfer learning model exhibited high performance, reaching 98.6% accuracy in classifying starch adulteration in chicken meat [38]. This high performance can be attributed to the unique roles of GoogLeNet network, which efficiently captured amplified spectral features from the CWT, while transfer learning effectively addresses the need for extensive computing resources or large datasets in CNN training and avoids the overfitting caused by small datasets. In addition, GANomaly was applied to honey fraud detection, achieving 100% accuracy in identifying diverse frauds, such as aged and syrup-adulterated honey (Figure 9d), with a training set consisting solely of pure honey samples [131]. Under identical conditions, these results outperformed conventional techniques such as DD-SIMCA and OC-PLS. Furthermore, HSI combined with DM has demonstrated broad applicability across diverse scenarios. Typically, this approach has facilitated the detection of adulteration (including pork or duck meat) in restructured beef [197], discrimination of analogous density foreign matter in soy protein meat [25], and discrimination of homochromatic foreign materials in cut tobacco [200], with accuracies exceeding 95%. However, the application of DL algorithms to small-sample datasets has usually suffered from overfitting. Encouragingly, transfer learning [38,39] and advanced parameter optimization methods, such as beluga whale optimization (BWO) [198] and Grey Wolf Optimizer (GWO) [99], have proven to alleviate this issue and enhance the training efficiency.

### 4.5. Heavy Metal Detection

**Figure 10:**
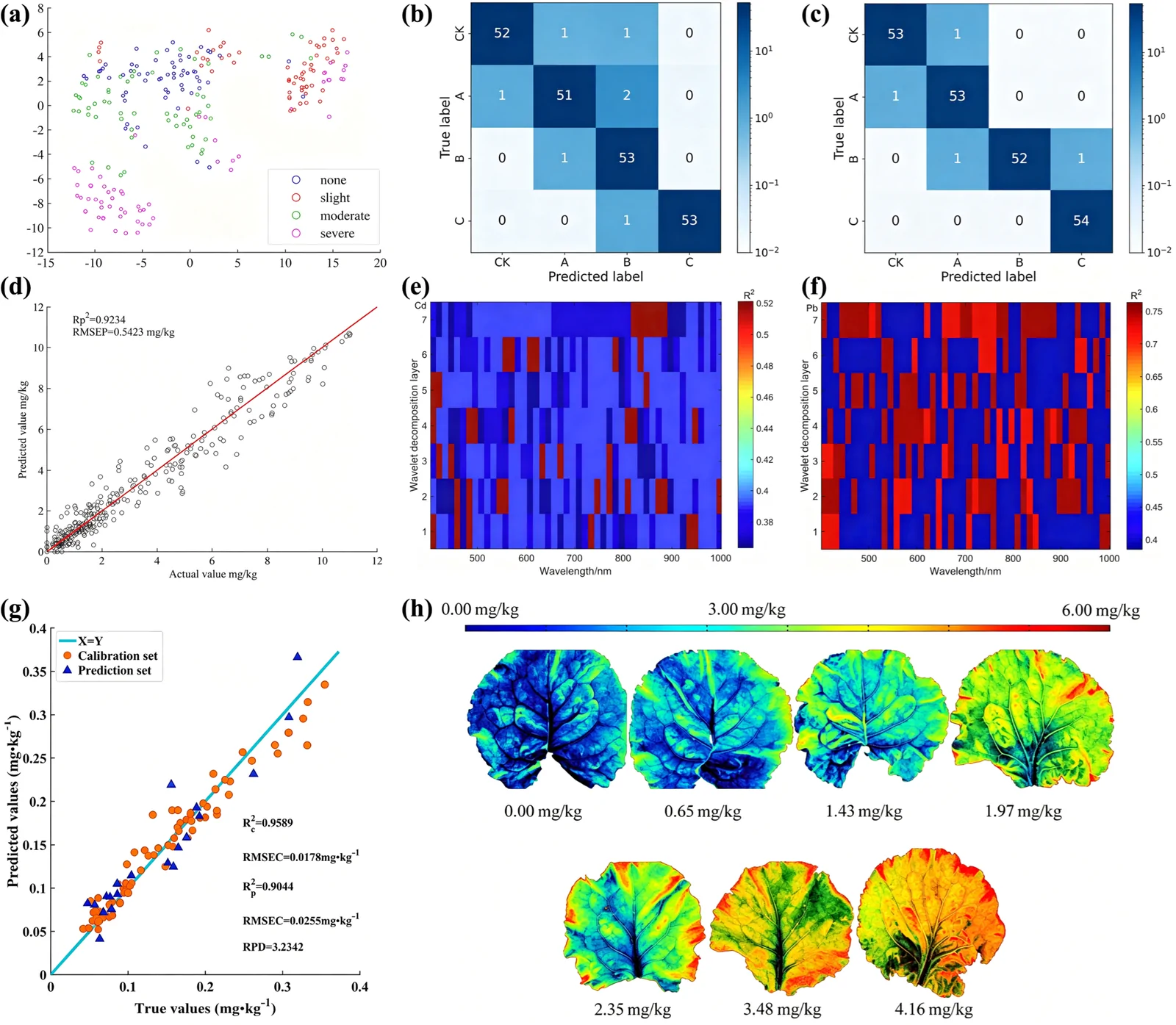
(**a**) Visualization map of feature data via CARS for detecting lead pollution levels (the average measured values are 0, 0.264, 0.549, and 1.128 mg/kg for none, slight, moderate, and severe) in lettuce leaves [197]. Confusion matrices of (**b**) ResNet-50 and (**c**) RegNetX-6.4GF models for copper stress level classification (CK: 0, A: 100, B: 300, and C: 500 mg/L) in oilseed rapes [201]. (**d**) Classification of cadmium content in lettuce leaves based on PSO-DBN models [202]. R^2^ values of (**e**) Cd and (**f**) Pb in lettuce [205]. The average measured contents of Cd and Pb in lettuce are (0.73, 0.77, 0.91, 1.85, 1.34, and 1.49 mg/kg) and (0.056, 0.069, 0.084, 0.19, 1.63, and 2.18 mg/kg), respectively. (**g**) Prediction of cadmium content in lettuce leaves [203]. (**h**) Distribution map of cadmium content in lettuce leaves [208].

In recent years, HSI combined with ML and DL algorithms has enabled comprehensive assessment of heavy metal pollution in foods, encompassing qualitative classification, quantitative regression, and spatial visualization analysis. Earlier studies predominantly relied on shallow ML algorithms, such as SVM and PLS-DA, in conjunction with feature selection techniques including CARS and IRIV. These approaches were used to classify heavy metal stress levels and to quantitatively predict contaminant concentrations. For instance, the SVM model achieved a classification accuracy of 93.49% on 1D spectral data for copper stress classification in oilseed rapes [201]. Moreover, the CARS-SVM model attained 91.67% accuracy on the test set for lead pollution identification in lettuce leaves (Figure 10a) [197]. In that study, CARS selected 71 informative wavelengths from 399 bands via Monte Carlo sampling and PLS regression coefficients, and the resulting features were fed into an SVM model with the penalty parameter and kernel function parameter optimized by grid search. These methods have reduced the dimensionality of HSI data; yet, their inherent linear or weakly nonlinear transformations are ineffective for extracting complex spectral features.

Notably, DL algorithms have enhanced both the automation of feature extraction and the predictive accuracy of modeling, especially in the synergistic utilization of 1D spectral sequences and 2D spatial features. Recently, 1D-CNN and 1D-residual networks (such as 1D-RegNetχ-6.4GF) have been employed to directly extract deep abstract features from raw spectra, achieving accuracies of 96.69% and 98.15% (Figure 10b,c), respectively, in oilseed rape copper stress classification [201]. The performance gap between 1D-CNN and 1D-RegNetχ-6.4GF demonstrates that the combination of precise feature learning and spatially enhanced inputs can extract discriminative features related to copper stress-induced chlorophyll degradation and cell structure damage. In addition, unsupervised pre-training models, such as SAE, DBN, and SDAE, have been extensively applied to quantitative prediction. Typically, the PSO-DBN model achieved an R^2^_P_ of 0.9234 and an RMSEP of 0.5423 mg/kg in detecting cadmium content in lettuce leaves (Figure 10d) [202]. In another study, SDAE-SVR achieved an R^2^ of 0.9273 in predicting cadmium content in oilseed rape leaves [203]. In addition, the WT-SDAE-SVR model yielded an accuracy of 0.9388 for predicting lead content in oilseed rape leaves [204]. Further advances have been made with more sophisticated spectral data. For instance, Stacked Convolutional Autoencoder (SCAE) combined with WT algorithm for extracting deep features, followed by SVR regression, achieved the R^2^ of 0.9319 and 0.9418 for the prediction of combined heavy metals (cadmium and lead) in lettuce (Figure 10e,f) [205]. Notably, the WT-SCAE hybrid model outperformed the single SCAE without WT (0.9176 for cadmium and 0.9281 for lead), confirming that multi-scale decomposition enhances the network’s capacity to disentangle overlapping spectral signatures of co-existing metals. In a separate study, the VMD-SAE hybrid model achieved an R^2^ of 0.9372 for lead content prediction in eggs [206]. Furthermore, ensemble learning strategies, such as stacking and blending, were applied for detection of cadmium content in oilseed rape leaves, with the stacking-RF model achieving an improved R^2^ (0.9815) [207]. Specifically, two-layer stacking and blending models were developed, where one layer used SVR, ELM, DT, and RF algorithms as a basic learner, and the other layer used SVR or RF as a meta-learner. These learners were used to train and predict on the training and validation sets. These approaches excel at extracting nonlinear and multi-scale spectral features related to heavy metals, offering a substantial advantage over conventional ML methodologies.

To improve the model generalizability and practical utility, investigations have recently focused on multi-source data fusion, transfer learning, and visualization analysis. In one report, deep feature fusion of fluorescence HSI and near-infrared spectra was employed for trace cadmium detection in lettuce leaves, where CNN-extracted features coupled with LSSVR achieved an R^2^ of 0.9044, significantly surpassing that based on individual spectral data (Figure 10g) [203]. The enhanced performance results from the fused spectral data providing a more comprehensive range of physiological and biochemical information than individual spectral data. In another study, the transfer stack denoising autoencoder (T-SDAE) algorithm enabled the discernment of cadmium content in oilseed rape leaves across environments with differing silicon levels, achieving an R^2^ of 0.9273 [203]. In addition, the Grad-CAM model revealed latent physiological changes in plants under heavy metal stress, guided feature learning within CNNs, and showed the effectiveness of false-color image construction in extracting discriminative features [201]. Meanwhile, distribution maps were further built for pixel-level visualization of cadmium content in lettuce leaves (Figure 10h) [208].

### 4.6. Mold Detection

**Figure 11:**
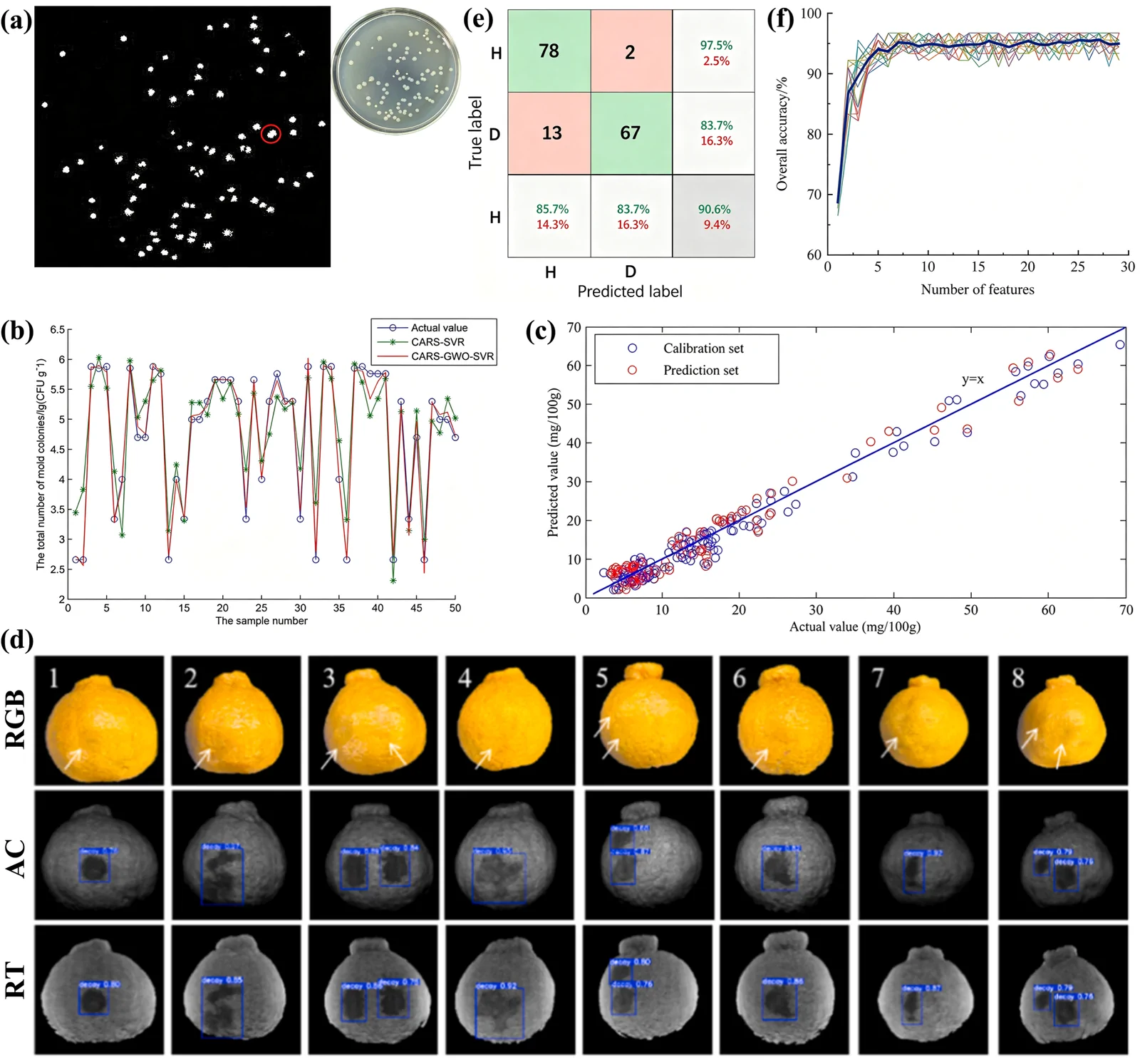
(**a**) Colony count using HSI techniques [210]. (**b**) Prediction of mold colonies in rice [211]. (**c**) Detection of solanine content in potatoes [212]. (**d**) Detection of decayed regions of dekopon by YOLOv11-SSConv models [24]. (**e**) Confusion matrix of the CNN model for the orange–mandarin mixed testing sets [23]. (**f**) Overall classification accuracy for detecting the early decay in oranges [213].

In recent years, substantial progress has been achieved in the applications of ML and DL algorithms to HSI for the detection of bacterial infections and harmful substances in food products. Conventional techniques (e.g., culturing and GC-MS) are time consuming and destructive, whereas HSI combined with ML and DL offers non-destructive and rapid early warning capabilities. Typically, Wang et al. employed a microfluidic chip to enrich *Botrytis cinerea* spores and used lens-free diffraction imaging [209]. Then, automated spore counting was realized through diffraction image processing and morphological feature extraction, yielding an average error of 6.42%. Shi et al. proposed a KNN classification model to tackle the interference of food debris on agar plates, whose color and shape resembled colonies [210]. The model effectively separated colonies from the background, leading to counts in strong agreement with manual counting with an accuracy of 0.9998 (Figure 11a). Cong et al. extracted characteristic wavelengths using CARS and subsequently optimized the SVR model through the Grey Wolf Optimization (GWO) method [211]. This strategy enabled high-precision prediction of total mold colonies in rice, yielding an R^2^ of 0.9511 on the prediction set (Figure 11b).

The practical utility of the HSI technique is further corroborated by diverse applications in mycotoxin and pathogen detection. Lu et al. employed fluorescence HSI to extract the bud eye region of potatoes as the region of interest (ROI) and developed an SVR model for solanine content prediction, achieving an R^2^ of 0.9143 on the prediction set (Figure 11c) [212]. Shen et al. employed the near-infrared HSI combined with the local PLS algorithm for quantitative detection of deoxynivalenol in single wheat kernels, featuring superior performance with an RPD of 2.24 [214]. Yang et al. applied line-scan Raman HSI coupled with the SVM model to identify Peanut Kernels infected with three different strains of *Aspergillus flavus* [215]. Their model achieved a binary classification (namely, healthy vs. *A. flavus*) with accuracy exceeding 99%, and a three-class classification with accuracy around 90%. Collectively, these studies suggest that HSI techniques combined with traditional ML algorithms (e.g., SVR, PLS, RF, and SVM) effectively extract spectral and spatial features for accurate qualitative and quantitative analysis of fungal infections, toxins, and harmful metabolites in foods.

In recent years, the introduction of structured illumination imaging and DL models has further enhanced both detection sensitivity and robustness for early-stage infections and diseases that present no obvious surface features. Luo et al. achieved 98.4% accuracy in early detection of decay in weakly fluorescent dekopon fruits by combining structured-illumination reflectance imaging with the YOLOv11-channel reconstruction convolution (SCConv) lightweight DL model (Figure 11d) [24]. This approach decreased parameters by around 4.8% and increased inference speed by 25% compared to the original YOLOv11. Notably, the parameter reduction and speed improvement are significant for practical applications, since they enable real-time processing on resource-constrained sorting lines without sacrificing detection sensitivity. In another report, Cai et al. combined visible LED structured-illumination reflectance imaging with the CNN model to detect decay in oranges and sugar mandarins [23]. The CNN built with Ratio images, which are the ratio of alternating component images to the corresponding direct component images, yielded 96.3% accuracy on orange–mandarin mixed validation sets (Figure 11e), significantly outperforming PLS-DA (89.6%). The performance gap highlights the CNN’s superior ability to capture spatially distributed texture patterns in Ratio images. In a later study, they introduced the spiral phase transform (SPT) algorithm, which demodulates the pattern images based on 1–2 phase-shift images [213]. By employing the Random Frog algorithm to extract the informative features and using the LSSVM model built with seven texture features from the Ratio images for classification, they achieved an accuracy of 95.1% for early orange decay detection (Figure 11f). Notably, this strategy reduces image acquisition time while maintaining high classification performance. In detecting internal disorders, multimodal imaging techniques have offered superior capability. Typically, Nie et al. demonstrated that soft X-ray imaging outperforms near-infrared spectroscopy for detecting black heart disease in pomegranates [71]. In detail, the RF model built on textural features extracted from the soft X-ray images achieved a prediction accuracy of 93.10%, outperforming the near-infrared spectroscopy-based predictive model, which yielded an R^2^ of 86.00%. In summary, ML and DL algorithms coupled with structured illumination, multi-spectral fusion, and feature optimization are driving HSI techniques toward practical applications, enabling rapid, non-destructive, and accurate detection of microbial pathogens and mycotoxins in various foods.

### 4.7. Others

Beyond the above applications, ML and DL algorithms have markedly advanced HSI technology in several other domains, such as seed viability assessment, pesticide residue detection in food products, and spatial distribution mapping of nutritional components. In seed viability assessment, Li et al. achieved notable progress for soybean seed viability identification by employing fluorescence HSI combined with the CARS-SVM-AdaBoost model, yielding 100% accuracy on both calibration and validation sets [216]. Similarly, Sun et al. utilized the visible near-infrared HSI technique combined with the PCA-ABC-SVM model, which also achieved an accuracy of 100% in watermelon seed viability detection [217]. These findings indicate that feature wavelength selection and intelligent optimization algorithms can effectively enhance model generalizability. In pesticide residue detection, various strategies for feature extraction and wavelength selection have been studied, such as chemical molecular structure coupled with wavelet transform (CMS-WT) [218], optimal wavelength identification with the WT-MD-MCCV algorithm [219], and feature selection methods such as CARS-SPA and RF-RFE-SPA [78]. Leveraging these methods, SVR and LSSVR models have achieved accuracies (R^2^_p_) for pesticide residue detection ranging from 0.82 to 0.94. Additionally, the SPA-MLR model was employed to visualize the spatial distribution of pesticide residues on mulberry leaves [220].

HSI has further been extended to the analysis of nutritional component distribution in food matrices. For instance, Shi et al. employed Genetic Algorithm-interval Partial Least Squares (GA-iPLS) and Genetic Algorithm-Partial Least Squares (GA-PLS) algorithms to establish quantitative models for protein, carbohydrate, and sialic acid content in edible bird’s nest, and further generated pixel-level distribution maps of these components [221]. In another study, Gao et al. adopted Detrended Fluctuation Analysis (DFA) preprocessing combined with the KNN classifier to construct pixel-wise segmentation maps for pizza toppings and analyzed the distributional uniformity by using Moran’s I [222]. This finding demonstrates the unique advantage of spectral–spatial fused information for evaluating the uniformity of complex foods.

Collectively, the deep integration of ML and DL algorithms with HSI technology has markedly advanced the quality assessment of agricultural and food products, progressing from traditional point-based measurements to area-scale imaging and spatially resolved quantification. This advancement supports rapid seed viability assessment and food safety and quality evaluation.

## 5. Challenges and Outlook

As shown above, ML and DL algorithm-assisted HSI techniques have made significant progress in food quality and safety detection in recent years. However, challenges remain. First, data scarcity remains a critical issue. HSI for food quality and safety detection yields massive, high-dimensional spectral data; however, annotating food samples is both time consuming and expertise dependent, which severely limits the availability of large-scale labeled datasets [223,224]. Moreover, the heterogeneity of food matrices poses a significant barrier to model generalization across various foods. Second, model interpretability and generalization are deficient. DL architectures, often known as *black boxes*, undermine their credibility in regulatory settings. Meanwhile, traditional chemometric models, such as PLSR and PCR, struggle to handle nonlinear and mixed spectral data [225]. Third, the translation of HSI technology to practical applications is constrained by both technical and economic factors, primarily the high computational cost of deep architectures and the lack of standardized online-inspection validation criteria, which hinder the application of HSI techniques. Additionally, calibration robustness and operational variability, both induced by changes in experimental conditions, including samples, instruments, and environments, remain persistent challenges.

To address these challenges, various strategies have been demonstrated. To mitigate data scarcity, transfer learning and few-shot learning, achieved via cross-category feature transfer, have been explored for reducing dependence on large-scale labeled datasets [224]. In addition, integrating data augmentation with few-shot learning provides opportunities to alleviate overfitting under small-sample data [226]. For model optimization, explainable AI techniques (typically, SHAP [227]) have been employed to interpret model predictions and enhance decision transparency. Lightweight model designs (for example, YOLOv11-SCConv) have been adopted to reduce computational burden while preserving accuracy [24]. Additionally, multimodal data fusion strategies have been employed to enhance the comprehensiveness and reliability of models [224,228]. To facilitate practical applications, diagnostic wavelength selection has been utilized to simplify complex HSI systems into multispectral systems [229]. This approach, combined with sensor miniaturization [227,230] and standardized data collection and validation [227], is expected to provide hardware foundations for industrial applications. Currently, the HSI technique primarily functions as a screening and triage tool rather than a standalone regulatory and confirmatory method. Its regulatory application depends on validated protocols, standardized performance metrics, and follow-up confirmation.

## 6. Conclusions

This review has comprehensively summarized recent advances in ML- and DL-assisted HSI for food quality and safety assessment. Traditional ML methods, such as PLSR, SVM, and RF, retain their value in small-sample, high-interpretability scenarios, offering robust solutions for quantitative prediction of quality and qualitative discrimination of varieties and origins. In contrast, DL algorithms, such as CNNs and LSTMs, have demonstrated superior feature learning, effectively overcoming manual feature extraction limitations and achieving remarkable accuracy in complex spectral features, such as early damage detection, adulteration detection, and heavy metal classification. The integration of ML and DL is expected to advance HSI toward practical applications, enabling rapid, non-destructive, and spatially resolved quality detection across diverse foods.

## Figures and Tables

**Table 1:** Typical hybrid DL models used in HSI food quality and safety detection.

Hybrid Models	Advantages	Typical Applications in Food Quality Detection
CNN-LSTM	CNN for local features, LSTM for sequential spectral dependencies.	Quality of freeze–thawed chicken [80], and SSC of fruits [36].
CNN-Gated Recurrent Unit (CNN-GRU)	CNN for local features, GRU for sequential spectral dependencies.	Quality of surimi gel [144].
CNN-DotGRU-SelfAttention (CDGSA-Net)	Improve model generalization across species.	Chilling damage of kiwifruits [147].
Multi-attention convolutional neural network (MA-CNN)	Enhances key features and weakens redundant information.	SSC of apples [148].
Gramian angular difference Fields-multi-task convolutional neural network (GADF-MTCNN)	Simultaneously detects multiple oxidation indicators.	Lipid and protein oxidation in frozen-thawed chicken meat [149].
Stacked Denoising Autoencoder-PLSR-Ridge Regression (SDAE-PLSR-RR)	Combines deep abstraction with linear interpretability.	SSC and color indices of apples [94].

## Data Availability

No new data were created or analyzed in this study. Data sharing is not applicable to this article.
